# Conditional knockout of membrane‐type I matrix metalloproteinase in smooth muscle cells of adult mice alleviates atherosclerosis without affecting basic cardiovascular function

**DOI:** 10.1002/ctm2.70739

**Published:** 2026-07-07

**Authors:** Suha Jarad, Hong‐Mei Gu, Daniel Huang, Peter Amadi, Govind Gill, Floor Spaans, Aakar Chatha, Ala Yousef, Murilo E. Graton, Raj Patel, John M. Seubert, Ying Wang, Gordon Francis, Xiao‐Dan Xia, Sandra T. Davidge, Da‐Wei Zhang

**Affiliations:** ^1^ Department of Biochemistry Group on Molecular and Cell Biology of Lipids University of Alberta Edmonton Canada; ^2^ Department of Pediatrics Group on Molecular and Cell Biology of Lipids University of Alberta Edmonton Canada; ^3^ Departments of Obstetrics/Gynecology University of Alberta Edmonton Canada; ^4^ Women and Children's Health Research Institute University of Alberta Edmonton Canada; ^5^ Department of Pathology and Laboratory Medicine University of British Columbia Vancouver Canada; ^6^ Centre for Heart Lung Innovation University of British Columbia Vancouver Canada; ^7^ Faculty of Pharmacy and Pharmaceutical Sciences University of Alberta Edmonton Canada; ^8^ Department of Pharmacology Faculty of Medicine and Dentistry University of Alberta Edmonton Canada; ^9^ Department of Medicine University of British Columbia Vancouver Canada; ^10^ Affiliated Qingyuan Hospital, Guangzhou Medical University, Qingyuan People's Hospital Guangzhou China; ^11^ Department of Physiology University of Alberta Edmonton Canada

**Keywords:** atherosclerosis, cardiovascular disease, cell dedifferentiation, MMP14, PDGF signalling, smooth muscle cell

## Abstract

**Background:**

Atherosclerotic cardiovascular disease (ASCVD) is the leading cause of morbidity and mortality worldwide. Despite effective lipid‐lowering treatments, substantial residual risks remain. In atherosclerosis, vascular smooth muscle cells (SMCs) undergo dedifferentiation, promoting disease progression. Membrane‐type I matrix metalloproteinase (MT1‐MMP/MMP14) promotes SMC dedifferentiation. However, the effect of inhibiting MMP14 in adults, particularly those with existing atherosclerotic plaques, is unclear.

**Methods:**

We developed an inducible conditional SMC‐specific MMP14 knockout mouse model. Cardiac and vascular function were assessed using echocardiography and wire myography, respectively. Atherosclerosis progression and regression were evaluated in *Ldlr* ^−/−^ mice with or without MMP14 deficiency. snRNA‐seq of the aortas from *Ldlr* ^−/−^ mice was performed to determine the effect on SMC populations.

**Results:**

MMP14 expression was elevated in SMCs within fibroatheroma compared with the pathological intima thickening in coronary aortas from patients with ASCVD. Conditional knockout of SMC MMP14 in adult mice did not change plasma cholesterol levels or basic cardiac and vascular function. However, atherosclerosis development was reduced, and the regression of existing plaques was enhanced in *Ldlr* ^−/−^ mice lacking SMC MMP14. snRNA‐seq revealed increased fibroblast‐like SMCs and reduced foam cell‐like SMCs in MMP14‐deficient *Ldlr* ^−/−^ mice compared to *Ldlr* ^−/−^ mice. Furthermore, SMC MMP14 deficiency decreased SMC proliferation and migration, accompanied by reduced platelet‐derived growth factor receptor (PDGFR) β levels and attenuated PDGF signalling.

**Conclusion:**

SMC MMP14 promotes atherosclerosis in adult mice, likely through reducing PDGF signalling and inhibiting SMC migration and proliferation.

## Highlights

- Expression of smooth muscle cell‐specific MMP14 increases with atherosclerosis progression in humans.
- Inducible conditional knockout of SMC‐MMP14 in adult mice does not affect cardiac or vascular function.
- Inducible conditional knockout of SMC‐MMP14 in adult *Ldlr^−/−^* mice decreases atherosclerosis progression, increases plaque stability and accelerates regression of established plaques.
- Inducible conditional knockout of SMC‐MMP14 in adult *Ldlr^−/−^* mice increases fibroblast‐like SMC and decreases foam cell‐like SMC populations.
- MMP14 deficiency in SMCs increases LRP1 levels, reduces PDGFRβ levels, and diminishes PDGF signalling.

## Graphical abstract

1. Expression of smooth muscle cell‐specific MMP14 increases with atherosclerosis progression in humans.
2. Inducible conditional knockout of SMC‐MMP14 in adult mice does not affect cardiac or vascular function.
3. Inducible conditional knockout of SMC‐MMP14 in adult *Ldlr^−/−^* mice decreases atherosclerosis progression, increases plaque stability and accelerates regression of established plaques.
4. Inducible conditional knockout of SMC‐MMP14 in adult *Ldlr^−/−^* mice increases fibroblast‐like SMC and decreases foam cell‐like SMC populations.
5. MMP14 deficiency in SMCs increases LRP1 levels, reduces PDGFRβ levels, and diminishes PDGF signalling.

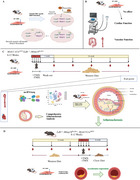

## 1 INTRODUCTION

Elevated plasma levels of low‐density lipoprotein cholesterol (LDL‐C) are causatively associated with increased risk of atherosclerotic cardiovascular disease (ASCVD), the leading cause of morbidity and mortality worldwide.^1^ Lipid‐lowering therapies, such as statins and proprotein convertase subtilisin/kexin type 9 (PCSK9) inhibitors, effectively reduce LDL‐C levels and lower the risk of ASCVD. However, substantial residual risk remains.^2^ The development and progression of atherosclerosis is driven by diverse factors^3^, ^4^, ^5^, ^6^, ^7^; therefore, combination therapies that target multiple pathways may be necessary to further decrease ASCVD risk.

Vascular smooth muscle cells (SMCs) are the primary cells in the arterial media and play a crucial role in maintaining arterial health. Under normal physiological conditions, SMCs maintain a differentiated phenotype characterized by low proliferation and synthetic activity but high expression of contractile proteins, such as α‐smooth muscle actin (α‐SMA). However, in atherosclerosis, SMCs dedifferentiate from a contractile to a synthetic phenotype, resulting in decreased expression of contractile proteins and increased proliferation and migration. SMCs migrate from the media into the intima, where they proliferate and undergo dedifferentiation into various phenotypes. SMCs alongside macrophages in the intima can take up modified lipoprotein particles, forming foam cells. Notably, SMC‐derived foam cells are substantial within atherosclerotic plaques, promoting disease severity.^3^, ^8^, ^9^, ^10^, ^11^ Thus, targeting SMC dedifferentiation presents a promising strategy for reducing ASCVD risk.

Membrane‐type I matrix metalloproteinase (MT1‐MMP/MMP14), encoded by the *Mmp14* gene, is a ubiquitously expressed zinc‐dependent endopeptidase. MMP14 is localized to specific areas within the cell membrane and plays diverse physiological and pathophysiological roles by degrading extracellular matrix (ECM) and non‐ECM components, as well as acting as a signalling protein.^12^, ^13^, ^14^ In atherosclerosis, MMP14 expression is upregulated within atherosclerotic plaques.^15^ Mice with *Mmp14* global knockout exhibit several growth and developmental abnormalities,^16^, ^17^ rendering it unfeasible for studying atherosclerosis. We and others have demonstrated that tissue‐specific deletion of MMP14 confers protection against atherosclerosis in mice. We have shown that MMP14 promotes the shedding of LDL receptor (LDLR).^18^, ^19^ Knockout of MMP14 in hepatocytes increases LDLR levels in the liver, lowering plasma LDL‐C levels and alleviating atherosclerosis in *Apoe^−/−^* mice.^18^ On the other hand, *Ldlr^−/−^* mice reconstituted with *Mmp14^−/−^* bone marrow develop more stable atherosclerotic plaques even though the overall plaque sizes are not significantly changed, indicating that macrophage MMP14 increases plaque vulnerability.^20^ MMP14 has also been shown to promote SMC migration and neointima formation, processes that promote lesion progression.^21^ Interestingly, constitutive embryonic deletion of SMC‐*Mmp14* induces aneurysm formation and exacerbates atherosclerosis in *Apoe^−/−^* mice.^22^ Of note, most patients with ASCVD requiring clinical intervention are adults with existing atherosclerotic lesions. However, the effect of MMP14 inhibition in adults on atherosclerosis, a more clinically relevant condition, is unclear.

Here, we showed that MMP14 levels are elevated in SMCs within fibroatheroma (AHA stage IV) compared with those in pathological intimal thickening (PIT, AHA stage III) in patients with ASCVD. Moreover, the deficiency of SMC MMP14 in *Ldlr^−/−^* mice fed a Western diet reduces atherosclerosis progression without aneurysm formation. In addition, conditional SMC‐*Mmp14* knockout alters SMC subpopulations in the aorta, increasing the fibroblast‐like SMCs and reducing foam cell‐like SMCs. SMCs with MMP14 deficiency display reduced SMC migration and proliferation, increased low‐density lipoprotein receptor‐related protein 1 (LRP1) levels, reduced platelet‐derived growth factor receptor (PDGFR) β levels, and attenuated PDGF signalling. Furthermore, SMC MMP14 deficiency in *Ldlr* ^−/−^ mice promotes regression of existing atherosclerosis without substantially changing plasma lipid levels.

## 2 METHODS

### 2.1 Human atherosclerotic lesion samples

Coronary lesions were obtained from 12 patients diagnosed with ischemic cardiomyopathy (*n* = 12) and one patient with dilated cardiomyopathy (*n* = 1). These tissues were formalin‐fixed and paraffin‐embedded (FFPE). H&E‐stained lesions were staged with a clinical pathologist based on the modified American Heart Association classification.^23^, ^24^ Seven of the 14 lesions were characterized as PIT, and the other seven were classified as fibroatheroma (Table S1). Human studies were approved by the Providence Health Care Research Ethics Board (H21‐01069) at the University of British Columbia. All coronary artery lesions were collected from explanted hearts donated by heart transplant patients. Informed consent was received from all patients through the Bruce McManus Cardiovascular Biobank at the Centre for Heart Lung Innovation, University of British Columbia. All study aspects adhered to the principles outlined in the Declaration of Helsinki.

### 2.2 Mice

*Ldlr^−/−^* and *Myh11‐*Cre^ERT2^ mice were purchased from The Jackson Laboratory (#019079). *Mmp14* ^flox/flox^ mice were generated as described previously.^18^ *Myh11‐*Cre^ERT2^/*Mmp14* ^flox/flox^ and *Ldlr^−/−^/ Mmp14* ^flox/flox^ mice were generated by crossing *Myh11‐*Cre^ERT2^ mice and *Ldlr^−/−^* mice with *Mmp14* ^flox/flox^ mice, respectively (Figure S1A). *Myh11‐*Cre^ERT2^/*Mmp14* ^flox/flox^/*Ldlr^−/−^* mice were generated by crossing *Myh11‐*Cre^ERT2^/*Mmp14* ^flox/flox^ mice with *Ldlr^−/−^* /*Mmp14* ^flox/flox^ (Figure S2A). All mice were bred at the University of Alberta animal facility. Male mice were used, as the *Myh11‐*Cre^ERT2^ transgene is located on the Y chromosome.^25^ All animal procedures were approved by the University of Alberta's Animal Care and Use Committee (Protocol#: AUP00003354) and were conducted in accordance with the guidelines of the Canadian Council on Animal Care and the NIH Guide for the Care and Use of Laboratory Animals.

Mice used were 8–12‐week‐old and fasted for 16 h before endpoint or blood collection, unless otherwise indicated. Mice were housed 2–5 per cage with free access to water in a temperature‐controlled facility (22°C, 43% humidity) with a 12 h light/dark cycle. After weaning, mice were fed a standard chow diet ad libitum until a Western diet (WD) was provided as per the study design. To induce conditional SMC‐*Mmp14* knockout, mice were administered tamoxifen (75 mg/kg) intraperitoneally for 5 consecutive days, followed by a 1‐week washout period. Control mice received a similar weight‐based vehicle (olive oil). Echocardiography, wire myography, and pressure myography were done in 1–2 weeks after the washout period.

Mice were euthanized by increasing carbon dioxide (CO_2_) concentration (flow rate at 30%–40% of chamber volume per minute) until the animals became unconscious with no respirations, followed by cervical dislocation. For anaesthesia, the animals were administered inhaled isoflurane, starting at 0.5% and gradually increasing by 0.5% increments every few breaths, up to a maximum of 4%. After full anaesthesia was confirmed, blood and tissue samples were collected.

For atherosclerosis studies, WD (Research Diets D12079Bi) containing 0.15% cholesterol (kcal from fat 40%, protein 16%, and carbohydrate 44%) was given for 16 weeks after the tamoxifen washout period. In an atherosclerosis regression study, mice were fed a WD for 12 weeks. Tamoxifen was then administered, and the mice were switched to a standard chow diet on the day after the last tamoxifen injection and continued on this diet for 6 weeks to allow plaque regression.

### 2.3 Echocardiography

Two‐dimensional transthoracic echocardiography was used to assess cardiac structure and function. Mice were anesthetized using 1%–2% isoflurane to maintain a heart rate between 400 and 500 beats/min. Echocardiography was performed on mice using the Vevo 3100 high‐resolution imaging system with a 40‐MHz transducer (MX550S; VisualSonics). The acquired images were analysed using the VisualSonics VevoLab software. M‐mode images obtained from the mid‐papillary short‐axis view were used to evaluate left ventricular internal dimensions and systolic function parameters, including ejection fraction, fractional shortening, cardiac output and stroke volume.

### 2.4 Wire myography

Mice thoracic and abdominal aortas were excised, isolated and cleaned from surrounding connective and adipose tissues in ice‐cold HEPES‐buffered physiological saline solution (PSS; in mmol/L: 10 HEPES, 142 NaCl, 4.7 KCl, 5.5 C_6_H_12_O_6_, 1.56 CaCl_2_, 1.17 MgSO_4_, 1.18 KH_2_PO_4_; pH 7.4), and then divided into 1.5–2 mm pieces, as previously described.^26^ The segments were then mounted on two 40 µm tungsten wires on a wire myograph system (620 M, Danish Myo Technology) and were kept in PSS (37°C). Data were recorded using LabChart software (version 8.1.13, ADInstruments). The aortas were normalized by stretching to a resting tension of 5 mN, and the PSS was replaced every 15 min during the 1 h stabilization period. Then, vessels were stimulated with a high potassium saline solution (KPSS; 123 mmol/L) to assess non‐receptor‐mediated smooth muscle cell function for up to 30 min or until plateau. Vessels were then stimulated once with phenylephrine (PE; 10 µmol/L) for 10 min, followed by exposure to methacholine (MCh; 3 µmol/L) for 2 min. Vessels were thrice washed and allowed to rest for 30 min, before performing a cumulative concentration response curve (CCRC) to PE (0.1 nmol/L—100 µmol/L), added in 2 min intervals or until plateau. Data were normalized to vessel length, and curves are presented as contractility (in mN/mm) and are summarized as area under the curve (AUC, in arbitrary units).

### 2.5 Pressure myography

Development of circumferential stress, as well as circumferential strain, was assessed by pressure myography (114P, Danish Myo Technology) using MyoVIEW4 software (Danish Myo Technology) and an inverted microscope (Axio Vert A.1, Zeiss) as described previously.^27^, ^28^ In brief, the mesenteric arcade was excised and placed in ice‐cold PSS. Then, mesenteric arteries (∼3–4 mm) were cleaned and cannulated on each side with two glass micropipettes, fixed with nylon thread, and kept in PSS (pH 7.4, 37°C). First, the intraluminal pressure was increased from 4 to 60 mmHg using 10 mmHg stepwise increments (at 5‐min intervals), and after 30 min, the intraluminal pressure was decreased to 4 mmHg (baseline). Then, the intraluminal pressure was increased from 4 to 160 mmHg (10 mmHg increments with 2‐min intervals) to assess the active mechanical properties of the vessel. The intraluminal pressure was returned to baseline (4 mmHg). Arteries were thrice washed and kept with EGTA‐Ca^2+^‐free PSS (in mmol/L: 142.0 NaCl, 4.7 KCl, 1.17 MgSO_4_, 1.18 KH_2_PO_4_, 10.0 HEPES, 2.0 EGTA) and incubated with papaverine (0.1 µmol/L) for 20 min to prevent the influx of calcium and to inhibit the release of intracellular calcium before increasing again the intraluminal pressure from 4 to 160 mmHg (10 mmHg increments with 2‐min intervals) to assess the passive mechanical properties of the vessels.^29^ The intraluminal pressure and vessel parameters (outer and inner diameters and wall thickness) were monitored and used for the calculation of the percentage development of myogenic tone, circumferential stress and circumferential strain.

### 2.6 Atherosclerosis assessment

At the endpoint, the aortas and hearts were collected immediately from euthanized mice after infusing with 100 units/mL Heparin Sodium in 1XPBS for 5 min at a rate of 2 mL/min and fixed in 4% paraformaldehyde overnight. The hearts were then embedded in optimum cutting temperature (OCT) embedding resin (eperedia^ET^ Cryomatrix^ET^). Serial sections (5 and 10 µm thick) were taken throughout the three aortic valves of each mouse, and sections were cut with cryo‐sectioning at the HistoCore facility at the Alberta Diabetes Institute, University of Alberta. Six aortic sinus sections per mouse (10 µm thick) were then stained with Oil Red‐O (ORO) as described previously.^30^, ^31^ Images were taken using an OMAX M837ZL‐C140U3 microscope. The atherosclerotic burden was quantified by measuring the surface area of ORO‐positive lesions in the aortic sinus. The lesion area was quantified using OMAX ToupView and expressed as the absolute area in the aortic sinus and as a percentage of the total aortic area, as described previously.^30^, ^31^ The area reported is averaged from six different sections per mouse and represented as one point.

### 2.7 Histology

Hearts collected from euthanized mice were fixed in 4% paraformaldehyde, embedded in paraffin (FFPE), cut into 8 µm thick sections throughout the three aortic valves, and stained with H&E at the HistoCore facility at the Alberta Diabetes Institute, University of Alberta. FFPE sections were deparaffinized and stained with Picro‐Sirius Red (PSR) staining according to the manufacturer's instructions. PSR staining was imaged by fiber birefringence under polarized light using a Leica THUNDER‐Deconvolution Widefield Microscope camera with a polarized lens (Cell Imaging Core, University of Alberta). Brightfield images were taken using an OMAX M837ZL‐C140U3 microscope. Collagen‐positive areas represented by the birefringence colour area relative to the lesion area in PSR pictures were quantified using ImageJ. The area reported is averaged from six different sections per mouse and represented as one point.

### 2.8 Immunofluorescence

FFPE sections of pathological intimal thickening and fibroatheroma samples were dewaxed in Citrisolve, and varying concentrations of isopropanol (100%, 90%, 70%). Antigen retrieval was performed using antigen retrieval buffer (10 mM sodium citrate, 0.05% Tween 20, pH 6.0), and the sections were heated for 22 min at 250°F. Frozen sections of the aortic valves of mice (5 µm thick) were bleached with 30% H_2_O_2_ and NaOH solution in PBS under LED light to eliminate autofluorescence prior to blocking.

FFPE sections were blocked with 10% goat serum in PBS for 1 h. In frozen sections, 0.4%Triton X‐100 in 1X PBS (PBS‐T) was used instead of PBS. The sections were incubated with primary antibodies, rabbit anti‐MMP14 (Abcam ab51074), rabbit anti‐CD68 (Abcam ab125212), mouse anti‐MYH11, rabbit anti‐Collagen type I (COL1) (Abcam ab270993), rabbit anti‐Fibronectin (FN) (Abcam ab268020), rabbit anti‐PPAR gamma (PPARG) (Abcam ab45036), mouse anti‐Lipoprotein lipase (LPL) (Abcam ab93898), or mouse anti‐αSMA (Sigma A5228), in 1% goat serum in PBS or PBS‐T overnight at 4°C. The samples were then washed with PBS or PBS‐T and incubated with Alexa Fluor anti‐mouse 488, anti‐rabbit 647, anti‐mouse 750, or anti‐rabbit 568 (Invitrogen) for 1 h at room temperature. The sections were washed with PBS or PBS‐T and incubated with DAPI for 10 min at room temperature. The samples were mounted using VECTASHIELD® Antifade Mounting Medium. A negative control without primary antibody was included for all samples. Images were captured using a Zeiss LSM880 (Carl Zeiss, Jena Germany) inverted confocal microscope with a 20x objective lens (University of British Columbia) or Zeiss Axioscan.Z1 Slide Scanner (Cell Imaging Core, University of Alberta). Fluorescence intensities of MMP14 overlapping with MYH11 fluorescence in the intima in human samples were quantified using ImageJ. αSMA^+^ cells were quantified relative to DAPI^+^ cells within the mouse lesions using QuPath® software.

### 2.9 Blood glucose and plasma lipid analysis

Blood samples from mice fasted for 16 h were collected into EDTA‐coated tubes and centrifuged at 2000 ×g for 20 min. Plasma was collected and analysed for triglycerides (TG) using a TG Kit (FIJIFILM Wako Diagnostics), and for total cholesterol (TC) using a Total Cholesterol E Kit (FIJIFILM Wako Diagnostics) according to the manufacturer's instructions. Blood glucose was measured using glucose test strips at the time of blood sample collection. Plasma lipoprotein profiles were analysed in the Lipidomics facility at the University of Alberta.

### 2.10 3‐D vascular smooth muscle cell explant culture

The thoracic aorta was excised from the aortas of *Myh11‐*Cre^ERT2^/*Mmp14* ^flox/flox^ mice after the washout period, carefully cleaned in 1XPBS, and dissected to remove periadventitial fat. Fragments of the thoracic aorta were then stripped of intima and adventitia, and the media of the vessel wall were dissected into 1 × 1 mm fragments. Media explants were then suspended within a solution of type I collagen gel medium (2.7 mg/mL; Enzo) prepared with NaOH on ice while still liquid as described previously,^21^ and were cultured for 8 days in 10 X DMEM medium supplemented with 10% FBS and a PDGF‐BB/FGF‐2 mixture (10 ng/mL each; GenScript). Explants were fixed for transmission electron microscopy (TEM) and processed at the Advanced Microscopy Facility at the University of Alberta. Images were acquired using a Philips/FEI Morgagni transmission electron microscope equipped with a Gatan camera. The distance between the SMC explant and adjacent collagen fibrils was quantified using OMAX ToupView software, with each data point representing the average of 10 measurements taken along the explant–collagen interface.

### 2.11 Single Nuclear RNA sequencing (snRNA‐seq)

The entire aortas collected from euthanized mice were immediately frozen in liquid nitrogen (*n* = 6 per group), and the frozen samples were shipped to IDseq Inc. for sample preparation and analysis. Nuclear isolation and library construction were conducted according to the 10X genomics reference genome version (refdata‐gex‐mm10‐2020‐A). Data were analysed using Dr. Tom software (IDseq). Cell clustering, Gene Ontology (GO), and Kyoto Encyclopedia of Genes and Genomes (KEGG) enrichment were performed using the Dr. Tom software that uses the Seurat Version 3.0.2. The first step in cell clustering was to calculate the principal components in the data. For in factor analysis approach, variables are grouped according to their correlation, so all variables in a specific group are highly correlated but poorly correlated with variables in other groups. The most significant principal components were screened based on the degree of enrichment and *p*‐value, combined with UMAP algorithm for nonlinear dimensionality reduction analysis and clustering cells into several types with parameter: FindClusters‐resolution 0.5. All sequencing data files are uploaded to the Gene Omnibus (GEO) public database under the accession number (GES320115).

### 2.12 Human aortic smooth muscle cells

Primary Human Aortic Smooth Muscle Cells (HASMCs, ATCC. PCS‐100‐012) were cultured in DMEM (high glucose, Sigma‐Aldrich) with 10% v/v fetal bovine serum (FBS) (Sigma‐Aldrich) in a humidified incubator at 37°C, 5% CO_2_. Cells used for experiments were at passages 2–8. Predesigned Dicer‐substrate DsiRNA against *MMP14* or negative siRNA (Table S2) were introduced into HASMCs using Lipofectamine™ RNAiMAX according to the manufacturer's protocol. 30–48 h post‐transfection, HASMCs were analysed.

### 2.13 Murine primary vascular smooth muscle cells

Murine primary SMCs were isolated from the thoracic aorta of 6–8‐week‐old *Myh11‐*Cre^ERT2^/*Mmp14* ^flox/flox^ mice receiving tamoxifen (conditional knockout of SMC *Mmp14*) or olive oil (control) after the washout period. The thoracic aorta was excised and carefully cleaned in 1XPBS and dissected to remove all periadventitial fat. The aorta was then incubated in a fresh enzyme solution (Collagenase type II, elastase, and soybean trypsin inhibitor in 1XHBSS) at 37°C, 5% CO_2_ for 10 min or until the adventitial layer was removed. Then, the aorta was washed with 1XPBS and cut open; the endothelial layer was gently removed by forceps. The aorta was cut into small pieces and incubated at 37°C (5% CO_2_) in a fresh enzyme solution until individual cells were seen. Enzyme activity was then stopped by adding 20% FBS in DMEM/F12. Suspended cells were spun for 5 min at 1000 × RPM. Cell pellets were then resuspended with 20% FBS, 1% Penicillin/Streptomycin/Amphotericin B in DMEM/F12 and plated on a cell culture plate. Medium was changed every three days. Cells used for experiments were at passages 2–8.

VSMC identity was validated by immunofluorescence and Western blot. Given that endothelial cell contamination is the most likely concern in this commonly used isolation protocol,^32^ we assessed the expression of the canonical VSMC marker (α‐SMA) together with the endothelial marker CD31. As shown in Figure S3, α‐SMA staining (green fluorescence) showed the expected elongated, filamentous cytoplasmic organization characteristic of SMCs, whereas CD31 staining (red fluorescence) was barely detectable. In addition, we performed Western blot analysis using lysates for the same cell preparations used in the following analyses (Figure S3). CD31 was assessed using human umbilical vein endothelial cell (HUVEC) lysate as a positive control to validate antibody efficacy. CD31 was undetectable in primary SMC lysate, while it was robustly detected in HUVEC lysate. Equal loading was verified by Vinculin. These results confirm the absence of detectable endothelial contamination in our isolated SMC populations.

For primary SMC experiments, cells were cultured in 6‐well plates until confluency. For PDGF treatment, cells were cultured in 6‐well plates until confluency, medium was then changed to 0.1% fatty acid‐free bovine serum albumin (BSA) in DMEM/F12 before PDGF treatment (10 ng/mL).

### 2.14 Adenovirus infection

Murine primary SMCs were isolated from *Mmp14* ^flox^ mice as described above; cells were cultured in 20% FBS in DMEM/F12 with 1% Antimicrobial/Antifungal in a 6‐well plate until 70% confluency. Cells were then starved in 0.1% BSA in DMEM/F12 with 1% Antimicrobial/Antifungal for 48 h before infecting them with 50 moi of AV‐Cre‐GFP or AV‐GFP (Vector Biolabs). RNA isolation was then performed 7 days following adenoviral infection.

### 2.15 Immunoblotting

Primary SMCs or HASMC (48 h post‐transfection) were collected for preparation of whole‐cell lysate using a RIPA lysis buffer (150 mM NaCl, 5 mM EDTA, pH 8, 50 mM Tris‐HCl, pH 8, 1% NP‐40, 0.5% sodium deoxycholate, 0.1% SDS) supplemented with cOmplete™ Mini, EDTA‐free protease inhibitor. Protein concentrations were determined using the BCA protein assay, and an equal amount of whole‐cell lysate was subjected to SDS‐PAGE, followed by electroblotting onto nitrocellulose membranes. The membranes were blocked with 5% non‐fat milk in 1X PBST at room temperature for 30 min and incubated overnight at 4°C with the following antibodies: A rabbit anti‐MMP14 monoclonal antibody (1:2000 dilution, Abcam ab51074), a mouse anti‐calnexin monoclonal antibody (1:10 000 dilution, Proteintech #66903‐1‐Ig), a rabbit anti‐vinculin monoclonal antibody (1:2000 dilution, Proteintech #66305‐1‐Ig), a rabbit anti‐LRP1 monoclonal antibody (1:20 000 dilution, NBP1‐40726), a rabbit anti‐PDGFRβ (1:2000 dilution, Proteintech #28992‐1‐AP), a mouse anti‐phosphorylated ERK (1:1000, Cell signalling #9106), and a rabbit anti‐total ERK (1:2000, Cell signalling #9102). Antibody binding was detected using goat anti‐mouse or anti‐rabbit IgG Alexa Fluor 680 or 790 secondary antibody (Invitrogen). The signals were detected and quantified on a Li‐Cor Odyssey Infrared Imaging System (Li‐Cor).

### 2.16 RNA isolation and Quantitative Real‐Time Polymerase Chain Reaction (RT‐qPCR)

Total RNA was isolated from primary SMCs or HASMC using the PureLink RNA Mini Kit (Invitrogen) according to the manufacturer's instructions. From tissues, total RNA was collected using TRIzol® according to the manufacturer's instructions. The High‐Capacity cDNA Reverse Transcription Kit was used to make the complementary DNA (cDNA). Relative gene expression to *GAPDH*, a housekeeping gene, was assessed by qRT‐PCR on a StepOnePlus™ system using SYBR^®^ Select Master Mix according to the manufacturer's instructions. The cycling procedure was set to 95°C for 10 min, followed by 40 cycles of 95°C for 15 s and 60°C for 1 min. The melting curve stage condition was: 95°C for 15 sec, 60°C for 1 min, and 95°C for 15 sec. Primers were designed by PrimerQuest Real‐Time PCR Design Tool, synthesized by IDT, Inc., and listed in Table S2. Each point represented in the graph is the average of three technical replicates per tissue per mouse or per condition.

### 2.17 Cell Counting Kit‐8 (CCK‐8)

HASMCs were seeded into a 96‐well plate with 5000 cells/well and transfected with negative control or *MMP14* siRNA. 30 h post‐transfection, medium was changed to 1% BSA in DMEM for 16 h to arrest the cell cycle. Medium was then changed to 10% FBS in DMEM for 24 h. After, 10 µL of CCK‐8 reagent was added to each well and incubated for 2 h. Absorbance at 450 nm was measured using a Spectramax 250 microplate reader. Primary SMCs isolated from mice were seeded at 10 000 cells/well in a 96‐well plate for 48 h. 10 µL of CCK‐8 reagent was then added. After a 2 h incubation, absorbance was measured at 450 nm. Primary SMCs were seeded at 10 000 cells/well in a 96‐well plate and transfected with negative control or *Lrp1* siRNA, 24 h post‐transfection, medium was changed to 0.1% BSA in DMEM/F12 with 1% Anti/Anti. 24 h later (48 h post‐transfection), medium was changed to 0.1% BSA/DMEM/F12/1% Anti/Anti containing 25 ng/mL PDGF‐BB, CCK‐8 was added, and absorbance was measured 4 h post PDGF‐BB.

### 2.18 Boyden chamber transwell‐assay

Primary SMCs or HASMCs (48 h post‐transfection) were trypsinized and counted. 50 000 cells in 250 µL of 0.1% BSA in DMEM or DMEM/F12 medium were placed on an 8 µm pore size insert pre‐coated with collagen type I. 500 µL of DMEM containing 20% FBS was placed below the insert. 24 h after, the insert was rinsed in 1X PBS, fixed, and then stained with crystal violet in 20% methanol as previously described.^19^ Cells on the top of the insert were removed using a cotton swab. Cells on the bottom of the insert were imaged on an OMAX M837ZL‐C140U3 microscope and counted (5 images per insert). Relative cell numbers were calculated by dividing the average cell numbers of the control group by the average cell numbers of each experimental group and expressed as percentage relative to the control group.

### 2.19 Statistical analysis

Prism GraphPad software (v10.0) (GraphPad Software) was used for statistical calculations. Data are represented as mean ± SD unless otherwise mentioned. Data normality was assessed using the Shapiro–Wilk test. Unpaired two‐tailed Student *t*‐test was used to compare continuous variables between 2 groups, for longitudinal and nested data, linear mixed effects model with mouse ID included as a random effect (REML) was used, while one‐way ANOVA test was used to compare 3 or more groups, followed by Tukey hoc pairwise tests. A two‐way ANOVA was used to analyse data with 2 factors, followed by Tukey post‐hoc analysis. Student's *t*‐test or ANOVA was used for statistical analysis only when normality was confirmed. *p* value ≤ .05 was considered statistically significant.

## 3 RESULTS

### 3.1 Characterization of SMC‐specific MMP14 conditional knockout mice

**FIGURE 1:**
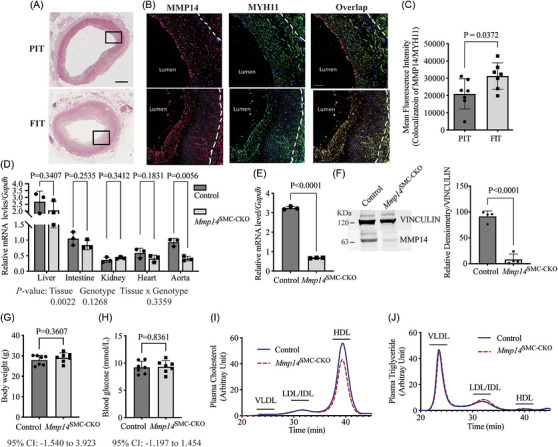
Expression of MMP14 and inducible SMC‐specific *Mmp14* knockout. (A) H&E staining of human coronary artery sections, pathological intimal thickening (PIT) and fibroatheroma (FIT) (*n* = 7/group, scale bar = 400 µm). (B) Immunofluorescence of the regions indicated with a square in (A). DAPI: blue; MMP14: purple; and MYH11: green (scale bar = 50 µm). (C) Quantification of the mean fluorescence intensity of MMP14 and MYH11 co‐localization. (D) Relative *Mmp14* mRNA level to *Gapdh* in different tissues of control and *Mmp14* ^SMCiKO^ mice (*n* = 3/group). (E) Relative *Mmp14* mRNA level to *Gapdh* in primary SMC isolated from the thoracic aortas of control and *Mmp14* ^SMC‐CKO^ mice (*n* = 3 /group). (F) MMP14 protein levels of primary SMCs and its quantification. The relative levels were determined by normalizing the densitometry of MMP14 to that of VINCULIN on the same membrane (*n* = 4/group). (G and H) Body weight (G) and blood glucose levels (H) of control and *Mmp14* ^SMC‐CKO^ mice (*n* = 7/group). (I and J) Fasting plasma cholesterol (I) and TG (J) profiles of control and *Mmp14* ^SMC‐CKO^ mice (*n *= 7/group). *n* refers to biological replicates. Data are represented as mean ± S.D. *p*‐value was calculated by unpaired two‐tailed Student's *t*‐test in panels (C) to (H). *p*‐value < .05 is considered significant.

It has been reported that MMP14 expression is notably high in human atherosclerotic plaques.^33^ Here, we assessed its levels in SMCs within PIT and fibroatheroma. The expression of MMP14 in MYH11‐expressing cells (a marker of SMCs) was evaluated in sections of coronary aortas from patients with ASCVD. As shown in Figure 1A–C, the expression of MMP14 in SMCs was significantly higher in fibroatheromas than in PIT sections, as evidenced by the colocalization of MMP14 with MYH11, suggesting that MMP14 expression increases with disease progression.

To investigate the potential effect of MMP14 inhibition in adults on atherosclerosis, we developed inducible SMC‐specific MMP14 conditional knockout mice. *Mmp14* ^flox^ mice, which have exons 2 and 4 of the *Mmp14* gene flanked by LoxP sites, have been used to generate hepatocyte‐specific MMP14 knockout mice in our previous study.^18^ *Mmp14* ^flox^ mice were bred with Myh11‐icre/ER^T2^ mice to generate *Mmp14* ^flox^/Myh11‐iCre/ER^T2^ mice (Figure S1A). The Myh11‐iCre/ER^T2^ transgene is inserted on the Y chromosome; therefore, only male mice were included in this study. Myh11‐iCre/ER^T2^ mice are commonly used to produce inducible SMC‐specific target gene knockout in mice.^10^, ^25^, ^34^, ^35^, ^36^ Genotyping confirmed the presence of floxed *Mmp14* and Cre in homozygous *Mmp14* ^flox^/Myh11‐iCre/ER^T2^ mice (Figure S1B). Adult male mice were then injected intraperitoneally with olive oil as control or tamoxifen (TAM) for 5 consecutive days to induce a conditional knockout of *Mmp14* in SMCs (*Mmp14* ^SMC^ *^−^* ^CKO^). mRNA level of *Mmp14* was substantially reduced in the aorta but not in other tissues of *Mmp14* ^SMC^ *^−^* ^CKO^ mice (Figure 1D). There was residual expression of *Mmp14* in the aorta, which might be attributed to other aortic cell types within the tissue. We then isolated primary SMCs from mouse thoracic aortas after the washout period. qRT‐PCR and Western blot analysis confirmed that mRNA and protein levels of MMP14 were markedly reduced in cells isolated from tamoxifen‐administered *Mmp14* ^flox^/Myh11‐iCre/ER^T2^ mice compared with mice receiving olive oil (Figure 1E,F), indicating an effective conditional knockout of MMP14 in SMCs of *Mmp14* ^SMC^ *^−^* ^CKO^ mice.

*Mmp14* ^SMC^ *^−^* ^CKO^ mice did not show a notable difference in size or appearance from control mice (Figure S1C). To estimate the completeness and specificity of recombination, genomic DNA was isolated from the aorta of WT mice, *Mmp14* ^flox^/Myh11‐iCre/ER^T2^ mice, olive oil‐treated *Mmp14* ^flox^/Myh11‐iCre/ER^T2^ (Control) mice, and tamoxifen‐treated *Mmp14* ^flox^/Myh11‐iCre/ER^T2^ (*Mmp14* ^SMC^ *^−^* ^CKO^) mice. A recombinant DNA band (∼700 bp) was detected exclusively in tamoxifen‐treated mice and was absent in all other groups, whereas the wild‐type allele was not observed in these samples (Figure S1D). SMC MMP14 deficiency also did not markedly alter body weight or blood glucose levels (Figure 1G,H). FPLC analysis of fasting plasma samples revealed comparable plasma cholesterol and triglyceride (TG) levels in most fractions of lipoprotein particles between the two groups, except for a slight reduction in HDL cholesterol in mice with SMC‐MMP14 deficiency (Figure 1I,J). Therefore, conditional knockout of MMP14 in SMCs of adult mice does not result in notable changes.

### 3.2 Effects of SMC *Mmp14* deficiency in adult mice on basic cardiovascular functions

**FIGURE 2:**
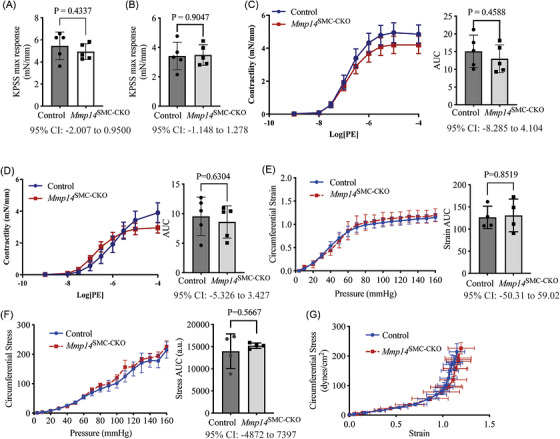
Effects of SMC‐specific *Mmp14* knockout in adult mice on vascular function. (A and B) Maximum vasoconstrictor capacity to high K^+^ physiological saline solution (KPPS) in thoracic (A) and abdominal aorta of control and *Mmp14* ^SMC‐CKO^ mice (*n* = 5/group). C and D, Vasoconstrictor response curve of the thoracic (C) and abdominal (D) aorta of control and *Mmp14* ^SMC‐CKO^ mice to phenylephrine (PE) and area under the curve (AUC) (*n* = 5/group). (E and F) Mesenteric artery circumferential strain (E) and stress (F) in response to increasing pressure and area under the curve (AUC) in control and *Mmp14* ^SMC‐CKO^ mice (*n* = 4/group). (G) Circumferential stress and strain in control and *Mmp14* ^SMC‐CKO^ mice (*n* = 4/group). *n* refers to biological replicates. Data are represented as mean ± S.D. *p*‐value was calculated by unpaired two‐tailed Student's t‐test in panels (A)to (F). *p*‐value < .05 is considered significant.

MMP14 cleaves ECM components, such as type I collagen (COLI) and fibronectin, and plays a key role in ECM remodelling and maintaining ECM homeostasis.^12^, ^21^ Therefore, we determined whether SMC *Mmp14* conditional knockout in adult mice affected mouse vascular function. The aorta and mesenteric arteries were isolated from control and *Mmp14* ^SMC‐CKO^ mice and subjected to wire myography and pressure myography, respectively. As shown in Figure 2A,B, SMC *Mmp14* deficiency did not significantly alter the maximal vasoconstrictor capacity of thoracic or abdominal aorta in response to high potassium physiological saline solution (KPSS), which assesses non‐receptor mediated smooth muscle cell function. Furthermore, the area under the curve (AUC) for the vasoconstriction responses to phenylephrine was also not notably affected in both the thoracic and abdominal aorta (Figure 2C,D). Moreover, no significant difference was seen in pEC50 and Emax of the thoracic or abdominal aorta between the groups (Figure S4A–D). To evaluate whether conditional knockout of SMC *Mmp14* affects vascular stiffness, we assessed the circumferential strain and stress and observed no marked difference between the two groups (Figure 2E–G). Furthermore, we employed echocardiography to assess cardiac function parameters, including heart rate, left ventricular ejection fraction, fractional shortening, cardiac output and stroke volume. No notable differences were observed between the two groups. Corrected left ventricular mass, internal wall measurement parameters, including interventricular septum (IVS), left ventricular posterior wall (LVPW), and left ventricular internal diameter (LVID) during diastole and systole, were also comparable between the two groups (Table 1). Therefore, conditional knockout of SMC *Mmp14* in adult mice does not notably affect basic cardiovascular function.

**TABLE 1:** Functional and structural results of echocardiogram.

Parameter	Control (*n* = 6)	*Mmp14* ^SMC‐CKO^ (*n *= 7)	*p‐*value
Heart Rate (beats/min)	457.45 ± 44.51	428 ± 44.36	0.899296
**Wall Measurements**			
Corrected LV mass, mg	118.85 ± 37.34	105.26 ± 33.45	0.969700
IVS‐diastole, mm	1.05 ± 0.22	1.08 ± 0.30	0.995776
IVS‐systole, mm	1.47 ± 0.26	1.52 ± 0.27	0.993843
LVPW‐diastole, mm	1.34 ± 1.09	0.89 ± 0.21	0.899296
LVPW‐systole, mm	1.45 ± 0.42	1.22 ± 0.22	0.899296
LVID‐diastole, mm	3.4 ± 1.24	3.27 ± 0.93	0.995776
LVID‐systole, mm	2.41 ± 0.56	2.37 ± 0.38	0.995776
**Cardiac Function**			
Ejection Fraction (%)	66.47 ± 4.18	63.07 ± 3.25	0.706367
Fractional Shortening (%)	36.32 ± 3.06	33.61 ± 2.26	0.628128
Cardiac Output ml/min	20.9 ± 3.13	16.17 ± 3.57	0.294907
Stroke Volume (µL)	45.64 ± 4.97	37.84 ± 7.11	0.404887

Male mice (10 weeks old) included six control mice and seven *Mmp14* ^SMC‐CKO^ mice. Results are presented as mean ± SD. An unpaired Student's *t*‐test was used to identify statistically significant differences between groups.

### 3.3 Effect of SMC *Mmp14* deficiency on atherosclerosis

Our next experiments aimed to evaluate whether SMC *Mmp14* deficiency in adult mice affected atherosclerosis. We generated *Ldlr* ^−/−^/*Mmp14* ^flox^/Myh11‐iCre^ERT2^ mice by crossing *Ldlr* ^−/−^/*Mmp14* ^flox^ mice with *Mmp14* ^flox^/Myh11‐iCre^ERT2^ mice (Figure S2A). Genotyping confirmed the absence of *Ldlr* and the presence of floxed *Mmp14* in *Ldlr* ^−/−^/*Mmp14* ^flox^ mice, as well as the presence of floxed *Mmp14* and *Cre* and the absence of *Ldlr* in *Ldlr* ^−/−^/*Mmp14* ^flox^/Myh11‐iCre/^ERT2^ mice (Figure S2B). Previous studies have shown that tamoxifen administration can reduce plasma cholesterol levels and affect the development of atherosclerosis in mice, either through 12‐day injections at 1.5 mg per day in corn oil or via a high‐fat/high‐cholesterol diet containing 15 µg tamoxifen/g food for 53 days.^37^, ^38^ We followed established protocols for tamoxifen‐inducible knockout in SMCs using Myh11‐iCreERT2 mice,^10^, ^25^, ^34^, ^35^, ^36^ administering tamoxifen in olive oil daily (75 mg tamoxifen/kg body weight) for 5 consecutive days. To assess whether this abbreviated tamoxifen administration affects plasma lipid levels and atherosclerosis development, we treated *Ldlr* ^−/−^/*Mmp14* ^flox^ mice with either tamoxifen or olive oil vehicle for 5 days, followed by 16 weeks of Western diet feeding (Figure S5A). Throughout a 16‐week period, both groups exhibited comparable body weight, blood glucose, plasma total cholesterol (TC), and plasma triglycerides (TG) (Figure S5B–E). Atherosclerotic plaque deposition in the aortic arches was also similar between groups (Figure S5F). Additionally, ORO staining revealed no marked difference in atherosclerotic plaques in aortic sinus sections (Figure S5G). Picrosirius Red (PSR) staining showed comparable intraplaque collagen content (Figure S5H). Together, these findings indicate that 5 days of tamoxifen administration do not significantly affect atherosclerosis development in *Ldlr^−/−^* mice.

**FIGURE 3:**
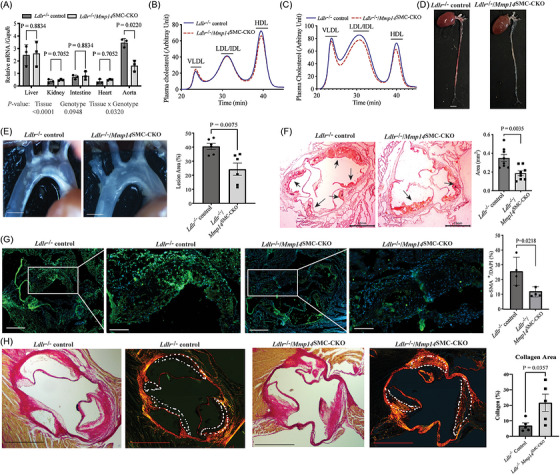
Effects of SMC‐specific *Mmp14* knockout in adult *Ldlr^−/−^* mice on atherosclerosis. (A) Relative *Mmp14* mRNA level to *Gapdh* in different tissues of *Ldlr* ^−/−^ control and *Ldlr* ^−/−^/*Mmp14* ^SMC‐CKO^ mice at the endpoint (*n* = 3/group). (B and C) FPLC of fasting plasma cholesterol of *Ldlr* ^−/−^ control and *Ldlr* ^−/−^/*Mmp14* ^SMC‐CKO^ mice at baseline (B) and the endpoint (C) (*n* = 6/group). (D) Representative images of the aortas of *Ldlr* ^−/−^ control and *Ldlr* ^−/−^/*Mmp14* ^SMC‐CKO^ mice (scale bar = 3 mm). (E) Representative images and quantification of lesions of the aortic arch of *Ldlr* ^−/−^ control and *Ldlr* ^−/−^/*Mmp14* ^SMC‐CKO^ mice (scale bar = 1 mm, *n* = 6/group). (F) Representative images and quantification of oil red O‐stained aortic sinus of *Ldlr* ^−/−^ control and *Ldlr* ^−/−^/*Mmp14* ^SMC‐CKO^ mice at the endpoint (*n* = 8/group, scale bar = 0.5 mm). (G) Immunofluorescence staining of DAPI (blue) and α‐SMA (green) in the aortic sinus of *Ldlr* ^−/−^ control and *Ldlr* ^−/−^/*Mmp14* ^SMC‐CKO^ mice (n = 4/group) at the endpoint (scale bar = 200 µm, inset picture scale bar = 100 µm). (H) Representative pictures and quantification of picrosirius red‐stained aortic sinus of *Ldlr* ^−/−^ control and *Ldlr* ^−/−^/*Mmp14* ^SMC‐CKO^ mice (*n* = 5/group, scale bar = 0.5 mm). Dashed white lines indicate plaques used for quantification. *n* refers to biological replicates. Data are represented as mean ± SD. *p*‐value was calculated by unpaired two‐tailed Student's *t*‐test in panels (A), and (E–H). *p*‐value < .05 is considered significant.

*Ldlr* ^−/−^/*Mmp14* ^flox^/Myh11‐iCre^ERT2^ mice then received olive oil (*Ldlr* ^−/−^ control) or tamoxifen to induce SMC MMP14 knockout (*Ldlr* ^−/−^/*Mmp14* ^SMC‐CKO^), followed by a WD feeding for 16 weeks (Figure S6A). qRT‐PCR confirmed efficient MMP14 SMC‐specific conditional knockout at the endpoint, revealing a significant reduction in aortic MMP14 expression in tamoxifen‐treated mice while expression in other tissues remained unchanged (Figure 3A). SMC MMP14 deficiency did not markedly affect plasma cholesterol (Figure 3B,C) or TG levels (Figure S6B,C) at baseline and the endpoint. Body weight and blood glucose levels were also comparable in the two groups (Figure S6D,E). In addition, we did not observe spontaneous aneurysm formation in any of the collected aortas in both groups at the endpoint (Figure 3D). On the other hand, fewer atherosclerotic plaques were observed in the aortic arch of *Ldlr* ^−/−^/*Mmp14* ^SMC‐CKO^ mice compared with those of *Ldlr* ^−/−^ control mice (Figures 3E and S7A). Hearts and entire aortas were then collected. Hearts were used for sectioning to assess atherosclerotic plaques, while aortas were used for immunoblotting, qRT‐PCR, and snRNA‐seq. As shown in Figure 3F, atherosclerotic plaques in the aortic sinus were substantially reduced in *Ldlr* ^−/−^/*Mmp14* ^SMC‐CKO^ mice compared with *Ldlr* ^−/−^ control mice (Figures 3F and S7B). To further assess the effects of tamoxifen and genotype on atherosclerosis progression, we employed a two‐way ANOVA to analyse aortic sinus lesions after 16 weeks of western diet feeding in both *Ldlr* ^−/−^/*Mmp14* ^flox^/Myh11‐iCre^ERT2^ and *Ldlr* ^−/−^/*Mmp14* ^flox^ mice treated with tamoxifen or olive oil (Figure S6F). Aortic sinus plaques were higher in *Ldlr* ^−/−^/*Mmp14* ^flox^ mice than in *Ldlr* ^−/−^/*Mmp14* ^flox^/Myh11‐iCre^ERT2^ mice, irrespective of tamoxifen administration, indicating a genotype effect. Notably, tamoxifen significantly reduced plaque sizes only in *Ldlr* ^−/−^/*Mmp14* ^flox^/Myh11‐iCre^ERT2^ mice compared with their olive oil‐treated counterparts. In contrast, *Ldlr* ^−/−^/*Mmp14* ^flox^ mice that lack Cre expression exhibited no difference between tamoxifen and olive oil treatments. These observations indicate that the effect of tamoxifen on atherosclerosis is Cre‐dependent and attributable to the conditional knockout of SMC MMP14.

Evaluating the cell content in aortic sinus sections showed a reduction in α‐SMA‐expressing cells within the plaques of *Ldlr* ^−/−^/*Mmp14* ^SMC‐CKO^ mice (Figure 3G). However, no significant differences were observed in necrotic core area, fibrous cap thickness, SMC cap index, or CD68^+^ cells between groups (Figure S6G–J). In contrast, PSR staining revealed increased collagen content within the plaques of *Ldlr* ^−/−^/*Mmp14* ^SMC‐CKO^ (Figures 3H and S7C). Therefore, silencing SMC *Mmp14* in adult *Ldlr* ^−/−^ mice reduced atherosclerotic lesion size and SMC content while increasing intraplaque collagen without affecting other parameters of plaque stability.

### 3.4 Effect of SMC MMP14 deficiency on cell populations

**FIGURE 4:**
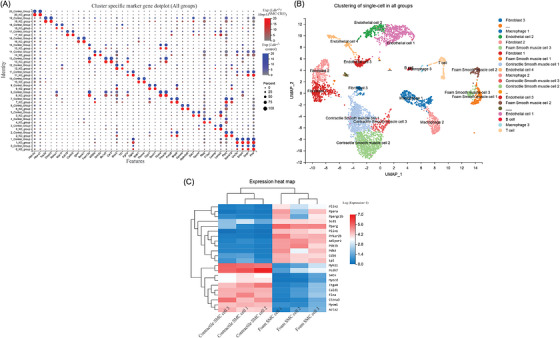
Single cell RNA seq analysis. (A) Dot blot showing the expression of the top two genes expressed in each cluster in the combined library of *Ldlr* ^−/−^ control and *Ldlr* ^−/−^/*Mmp14* ^SMC‐CKO^ groups. (B) UMAP analysis showing cells from the combined library of *Ldlr* ^−/−^ control and *Ldlr* ^−/−^/*Mmp14* ^SMC‐CKO^ groups coloured by cell cluster. (C) Heat map showing the expression of contraction and differentiation related genes, and dedifferentiation and lipid metabolism genes in the contractile and foam cell‐like SMC clusters.

SMCs within atherosclerotic plaques exhibit substantial heterogeneity, comprising various subpopulations with distinct functional characteristics. To investigate whether the distribution of these SMC subpopulations was altered, we performed snRNA‐seq on entire aortic tissues isolated from *Ldlr* ^−/−^ control and *Ldlr* ^−/−^/*Mmp14* ^SMC‐CKO^ mice fed a WD for 16 weeks (Figure S6A). Samples were pooled from six mice in each group. The total number of cells identified was 8839 cells in *Ldlr* ^−/−^ control group and 8846 cells in *Ldlr* ^−/−^/*Mmp14* ^SMC‐CKO^ group, filtered to 7416 and 7394 cells, respectively. Untargeted cell clustering revealed around 14 distinct cell clusters in each group, including SMCs, endothelial cells, fibroblasts, and different immune cells (including macrophages, T cells and B cells) (Table 2). The *Ldlr* ^−/−^ control group included four SMC clusters, two fibroblast clusters, three macrophage clusters, three endothelial cell clusters, two CD8+ T‐cell clusters and one B‐cell cluster (Figure S8A). The *Ldlr* ^−/−^/*Mmp14* ^SMC‐CKO^ group also exhibited four SMC clusters, two fibroblast clusters, and three macrophage clusters, but had four endothelial cell clusters and one T‐cell cluster (Figure S8B). The top 10 most significant marker genes expressed in each cluster (average logFC ranks the top 10 genes) in each group are shown in Figure S9A,B, while the top 2 most significant marker genes for each cluster are presented in Figure S10A,B. We also generated an integrated dataset by combining cells in both groups and re‐clustered cells together using the same top 10 genes (Figures S11 and 4A). This combined untargeted analysis revealed six SMC clusters, four endothelial cell clusters, three fibroblast clusters, three macrophage clusters, one T‐cell cluster and one B‐cell cluster for both groups (Table 3 and Figure 4B).

**TABLE 2:** Sn‐RNAseq cell clusters in *Ldlr* ^−−^ control and *Ldlr* ^−−^/*Mmp14* ^SMC‐CKO^ group.

Cluster Number	Cluster Cell Type	Cluster Number	Cluster Cell Type
***Ldlr* ^−/−^ Control Group**		***Ldlr* ^−/−^/*Mmp14* ^SMC‐CKO^ Group**	
**0**	Contractile Smooth muscle cell 1	**0**	Contractile Smooth muscle cell 1
**1**	Endothelial cell 1	**1**	Fibroblast 1
**2**	Contractile Smooth muscle cell 2	**2**	Contractile Smooth muscle cell 2
**3**	Fibroblast 1	**3**	Endothelial cell 2
**4**	Foam Smooth muscle cell 1	**4**	Macrophage 2
**5**	Macrophage 2	**5**	Macrophage 1
**6**	Endothelial cell 3	**6**	Foam Smooth muscle cell 1
**7**	Macrophage 1	**7**	T‐cell
**8**	Endothelial cell 2	**8**	Fibroblast 2
**9**	Macrophage 3	**9**	Endothelial cell 3
**10**	CD8+ T‐cell 1	**10**	Endothelial cell 1
**11**	Foam Smooth muscle cell 2	**11**	Endothelial cell 4
**12**	Fibroblast 2	**12**	Foam Smooth muscle cell 2
**13**	B‐cell	**14**	Macrophage 3
**14**	CD8+ T‐cell 2		

*Note*: Aortic samples were collected and pooled from six mice in each group.

**TABLE 3:** Sn‐RNAseq cell clusters in all groups.

Cluster Number	Cluster Cell Type
**0**	Contractile Smooth muscle cell 1
**1**	Contractile Smooth muscle cell 2
**2**	Endothelial cell 1
**3**	Fibroblast 1
**4**	Macrophage 1
**5**	Fibroblast 2
**6**	Macrophage 2
**7**	Endothelial cell 2
**8**	Foam Smooth muscle cell 1
**9**	Endothelial cell 3
**10**	T‐cell
**11**	Contractile Smooth muscle cell 3
**12**	Endothelial cell 4
**13**	Foam Smooth muscle cell 2
**14**	Fibroblast 3
**15**	Foam Smooth muscle cell 3
**16**	B‐cell
**19**	Macrophage 3

Within the SMC population, three clusters retained a contractile phenotype, characterized by high expression of *Myh11*, *Acta2*, *Tagln* and *Smtn*. The remaining three clusters showed downregulation of contractile markers and upregulation of genes associated with phenotype switching in SMCs, including *Pparg*, *Ppara* and *Lpl*. Notably, these SMC clusters upregulated numerous genes involved in lipid metabolism and uptake, suggesting a foam cell‐like SMC phenotype (Figure 4C).

**FIGURE 5:**
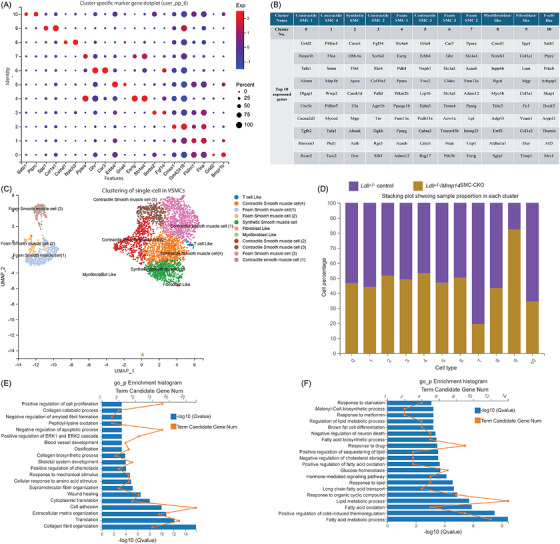
Single cell RNA seq analysis. (A) Dot blot showing the expression of the top two genes expressed in each cluster in the SMC clusters. (B) Table shows the expression of the top 10 genes in each cluster in the SMC clusters. (C) UMAP analysis showing cells from the SMC clusters coloured by cell cluster. (D) Stacking blot showing the proportion of cells from *Ldlr* ^−/−^ control and *Ldlr* ^−/−^/*Mmp14* ^SMC‐CKO^ group within each SMC cluster. (E) Go‐pathway enrichment analysis for the upregulated genes in the fibroblast‐like SMC cluster. (F) Go‐pathway enrichment analysis for the upregulated genes in the foam cell‐like SMC cluster.

To further dissect SMC phenotypic diversity, we refined the SMC clusters using marker genes shown in Figure 5A. The top 10 most significant marker genes expressed in each cluster (average log2FC ranks the top 10 genes) are shown in Figure 5B. We identified eleven different subclusters, including four contractile clusters, three foam cell‐like clusters, one synthetic cluster, one myofibroblast‐like cluster, one fibroblast‐like cluster and one T cell‐like cluster (Figure 5C, Table 4). Detailed analysis of SMC distribution across these subpopulations revealed that the *Ldlr* ^−/−^/*Mmp14* ^SMC‐CKO^ group exhibited a lower proportion of foam cell‐like SMCs and a higher proportion of fibroblast‐like SMCs than the *Ldlr* ^−/−^ control group (Figure 5D). As expected, fibroblast‐like SMCs were enriched in pathways associated with collagen synthesis and organization, while foam cell‐like clusters showed enrichment of pathways related to lipid uptake and metabolism (Figure 5E,F). To further validate these conclusions, we assessed the expression of collagen type I, fibronectin, PPAR‐γ, and LPL within the lesions of *Ldlr* ^−/−^ control and *Ldlr* ^−/−^/*Mmp14* ^SMC‐CKO^ mice. Expression of collagen type I and fibronectin was higher in *Ldlr* ^−/−^/*Mmp14* ^SMC‐CKO^ SMCs than in *Ldlr* ^−/−^ controls (Figure S12A,B), while the expression of PPAR‐γ and LPL was lower (Figure S12C,D). These shifts in SMC subpopulations in *Ldlr* ^−/−^/*Mmp14* ^SMC‐CKO^ mice favour phenotypes associated with the mitigation of atherosclerotic burden, consistent with the observed reduction in plaque size and increase in collagen content.

**TABLE 4:** Sn‐RNAseq cell clusters in SMCs.

SMC Cluster Number	Cluster Cell Type
**0**	Contractile Smooth muscle cell 1
**1**	Contractile Smooth muscle cell 4
**2**	Synthetic Smooth muscle cell
**3**	Contractile Smooth muscle cell 2
**4**	Foam Smooth muscle cell 1
**5**	Contractile Smooth muscle cell 3
**6**	Foam Smooth muscle cell 3
**7**	Foam Smooth muscle cell 2
**8**	Myofibroblast Like
**9**	Fibroblast Like
**10**	T‐cell Like

### 3.5 Effect of SMC MMP14 deficiency on SMC dedifferentiation

**FIGURE 6:**
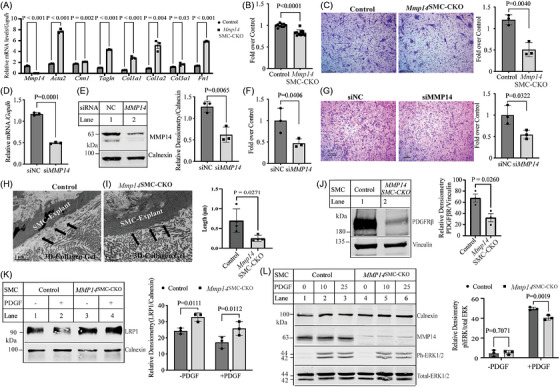
Effects of MMP14 deficiency on SMC proliferation and migration. (A) Relative mRNA levels of *Acta2*, *Cnn1*, *Tagln*, *Col1a1*, *Col1a2*, *Col3a1*, *Fn1* and *Mmp14* to *Gapdh* in primary SMCs isolated from control and *Mmp14* ^SMC‐CKO^ mice (*n* = 3). B, Proliferation of primary SMCs isolated from control and *Mmp14* ^SMC‐CKO^ mice (*n* = 10). (C) Representative pictures and quantification for primary SMCs migrated through collagen type I‐coated transwell (*n* = 3, scale bar = 0.5 mm). (D) Relative *Mmp14* mRNA expression over *Gapdh* in HASMCs transfected with control or MMP14 siRNA (*n* = 3). (E) MMP14 levels and its relative levels, which was normalized to CALNEXIN on the same membrane, in HASMCs (*n* = 3). (F) Proliferation of HASMCs transfected with control or siMMP14 (*n* = 3). (G) Representative pictures and quantification of control and siMMP14 HASMCs migrated through collagen type I‐coated transwell (*n* = 3, scale bar = 100 µm). (H and I) Electron microscopy picture and quantification of the clear area of control explant (H) and *Mmp14* ^SMC‐CKO^ explant (I) cultured in type I collagen gel (scale bar = 1 µm). (J) PDGFβ‐R protein expression and its quantification, which is normalized to Vinculin on the same blot in primary SMCs. (K) LRP1 protein expression and its quantification, which is normalized to calnexin on the same blot, in primary SMCs treated with or without PDGF‐BB (25 ng/mL) for 48 h. (L) phosphorylated ERK1/2 protein expression and its quantification, which is normalized to total ERK1/2 on the same blot, in primary SMCs treated with or without PDGF‐BB (10 or 25 ng/mL) for 10 min. n refers to biological replicates. Data are represented as mean ± SD. *p*‐value was calculated by unpaired two‐tailed Student's *t*‐test in panels (A) to (J). And by two‐way ANOVA followed by Tukey post‐hoc analysis in panels K and L. *p*‐value < .05 is considered significant.

We then investigated whether MMP14 deficiency affects SMC dedifferentiation. Primary SMCs were isolated from the aortas of *Mmp14* ^flox^/Myh11‐iCre/ER^T2^ mice administered tamoxifen (*Mmp14* ^SMC‐CKO^) or olive oil (Control). We observed that the mRNA levels of genes associated with differentiated contractile SMCs, including *Acta2*, *Tagln*, *Col1a1*, *Col1a2*, *Col3a1* and *Fn1*, were increased in SMCs with MMP14 deficiency (Figure 6A), indicating more collagen‐producing SMCs. To further confirm these findings, we used adenoviral Cre (AV‐Cre) to infect primary SMCs isolated from Mmp14^flox^ mice and induce *Mmp14* conditional knockout. SMCs infected with AV‐Cre had approximately 60% reduced *Mmp14* levels, which was associated with increased expression of *Acta2*, *Tagln* and *Col1a1* (Figure S13A). We next assessed cell proliferation and migration, features of SMC dedifferentiation. Cell migration was determined using a transwell assay, while proliferation was determined using the CCK‐8 assay. Primary SMCs with MMP14 deficiency exhibited a substantial reduction in proliferation (Figures 6B and S13B) and migration compared with control SMCs (Figures 6C and S13C). To further validate these observations, we knocked down MMP14 in cultured primary human aortic SMCs (HASMCs) using siRNA. qRT‐PCR and Western blot analysis confirmed efficient MMP14 knockdown (Figure 6D,E). Silencing *MMP14* markedly reduced HASMC proliferation and migration (Figures 6F,G and S13D,E). Therefore, the absence of MMP14 suppresses features of SMC dedifferentiation, including proliferation and migration.

SMC migration from the media requires degradation of the ECM, particularly the collagen I/III‐rich internal elastic lamina barrier separating the media from the intima.^3^, ^8^ MMP14 is a well‐established collagenase capable of cleaving both type I and III collagen.^12^ Thoracic aortic explants isolated from the aortas of control or *Mmp14* ^SMC‐CKO^ mice were embedded in a 3D collagen type I matrix. In explants from control mice, we observed a clear space near the tissue (Figures 6H and S14A), indicating active collagen lysis. In contrast, collagen surrounding explants from conditional SMC‐*Mmp14* knockout mice remained largely intact (Figures 6I and S14B), indicating impaired collagen degradation. In addition, previous studies have shown that LRP1 inhibits PDGF‐induced SMC dedifferentiation and that MMP14 promotes LRP1 processing.^39^, ^40^ Therefore, we examined PDGFRβ and LRP1 levels in isolated primary SMCs. We found reduced PDGFRβ levels in MMP14‐deficient primary SMCs compared with SMCs from control mice (Figure 6J). On the other hand, as shown in Figure 6K, SMCs from conditional SMC‐*Mmp14* knockout mice displayed increased LRP1 levels in the absence (lane 3 vs. 1) and presence of PDGF‐BB (lane 4 vs. 2). Furthermore, PDGF‐BB treatment resulted in less phosphorylated ERK1/2 in MMP14‐deficient primary SMCs while having no notable effect on total ERK1/2 levels compared with the control group (Figure 6L, lanes 5 & 6 vs. 2 &3). To further assess the role of LRP1 in the altered phenotypes of MMP14‐deficient VSMCs, LRP1 knockdown was performed in control and *Mmp14* ^SMC‐CKO^ primary SMCs (Figure S15A). *Mmp14* ^SMC‐CKO^ primary SMCs had significantly reduced proliferation compared with control cells. *Lrp1* knockdown increased proliferation in control SMCs but did not rescue proliferation in Mmp*14* ^SMC‐CKO^ primary SMCs (Figure S15B), while migration was unaffected in both groups (Figure S15C). Together, these findings suggest that MMP14 promotes SMC dedifferentiation. Its deficiency reduces PDGFRβ levels and attenuates PDGFRβ signalling, while increasing LRP1 levels. However, LRP1 does not appear to be essential for mediating the effect of MMP14 deficiency on SMC phenotypic changes.

### 3.6 Effects of SMC Mmp14 deficiency on atherosclerosis regression

**FIGURE 7:**
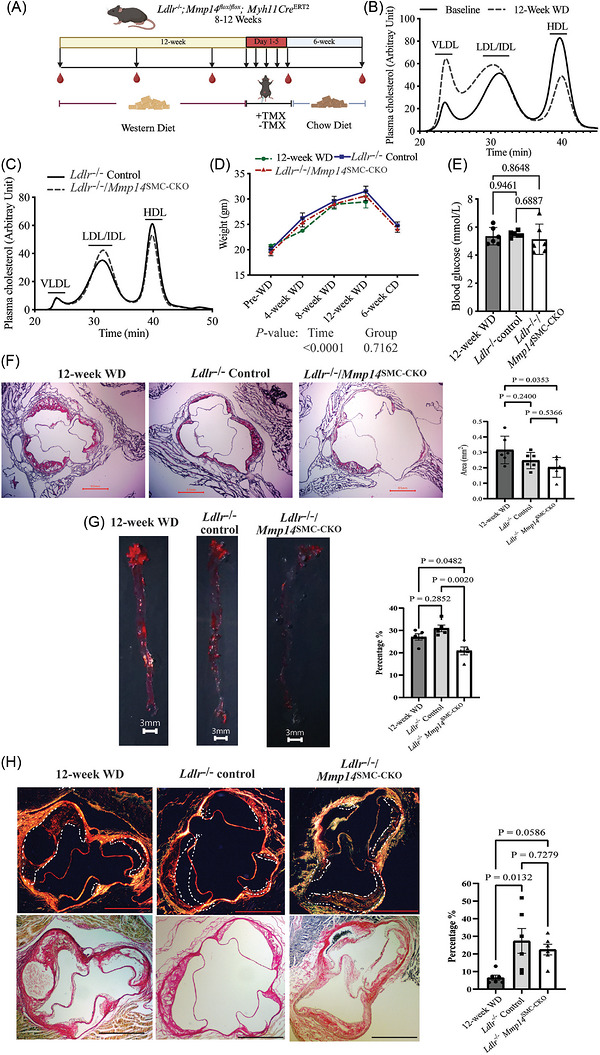
Effects of SMC‐specific *Mmp14* conditional knockout on atherosclerosis regression. (A) Graphical illustration of the study design. 10–12‐week *Ldlr^−/^* ^−^/*Mmp14* ^flox^/*Myh11*‐Cre male mice were fed a Western Diet (WD) for 12 weeks, followed by administration of tamoxifen or olive oil daily for 5 consecutive days. Mice were then fed a regular chow Diet for 6 weeks before euthanasia and tissue collection. (B) FPLC of fasting plasma cholesterol of *Ldlr^−/^* ^−^/*Mmp14* ^flox^/*Myh11*‐Cre mice at baseline and after a 12‐week WD feeding (*n* = 6/group). (C) FPLC of fasting plasma cholesterol of *Ldlr* ^−/−^ control and *Ldlr* ^−/−^/*Mmp14* ^SMC‐CKO ^mice at endpoint (*n* = 6/group). (D) Body weight of mice at baseline throughout the study period (*n* = 6 mice per group). (E) Blood glucose of baseline, *Ldlr* ^−/−^ control and *Ldlr* ^−/−^/*Mmp14* ^SMC‐CKO^ mice at endpoint (*n* = 5–6/group). F, Representative images and quantification of oil red O‐stained aortic sinus of baseline, *Ldlr* ^−/−^ control and *Ldlr* ^−/−^/*Mmp14* ^SMC‐CKO ^mice at the endpoint (n = 5‐6/group, scale bar = 0.5 mm). (G) Representative pictures and quantification of oil red O‐stained aortas of baseline, *Ldlr* ^−/−^ control and *Ldlr* ^−/−^/*Mmp14* ^SMC‐CKO^ mice at the endpoint (*n* = 5/group, scale bar = 3 mm). (H) Representative pictures and quantification of picrosirius red‐stained aortic sinus of baseline, *Ldlr* ^−/−^ control and *Ldlr* ^−/−^/*Mmp14* ^SMC‐CKO^ mice at the endpoint (*n* = 3–4/group, scale bar = 0.5 mm). *n* refers to biological replicates. Data are represented as mean ± SD. *p*‐value was calculated by one‐way ANOVA test followed by a Tukey hoc pairwise test in panels (E) to (H). *p*‐value < .05 is considered significant.

ASCVD patients requiring clinical intervention typically have existing atherosclerotic plaques. Taking advantage of our inducible gene knockout system, we investigated the effect of conditional SMC *Mmp14* knockout on atherosclerosis regression. *Ldlr* ^−/−^/*Mmp14* ^flox^/Myh11‐iCre^ERT2^ mice were fed a WD for 12 weeks to develop atherosclerosis (Figure 7A). A 12‐week WD feeding markedly increased plasma cholesterol levels (Figure 7B). A cohort of mice were collected at the end of WD feeding to assess the baseline atherosclerotic plaques. The remaining mice then received olive oil (*Ldlr* ^−/−^ control) or tamoxifen (*Ldlr* ^−/−^/*Mmp14* ^SMC‐CKO^), followed by a regular chow diet for 6 weeks (Figure 7A). To control for any potential confounding effects of tamoxifen on atherosclerosis regression, the same study design was applied to *Mmp14* ^flox^/*Ldlr* ^−/−^ mice. No significant difference was observed between mice with or without tamoxifen administration in body weight, blood glucose levels, plasma TC, plasma TG, or ORO‐stained aortic sinus sections at the endpoint (Figure S16A–E). These findings indicate that tamoxifen administration does not have a significant effect on atherosclerosis regression in *Ldlr* ^−/−^ mice.

At the endpoint, mice in *Ldlr* ^−/−^ control and *Ldlr* ^−/−^/*Mmp14* ^SMC‐CKO^ groups showed similar levels of plasma cholesterol (Figure 7C), as well as body weight and blood glucose levels (Figure 7D,E). ORO staining of aortic sinus sections revealed notably smaller plaques in *Ldlr* ^−/−^/*Mmp14* ^SMC‐CKO^ mice compared to both baseline and *Ldlr* ^−/−^ control groups, while *Ldlr* ^−/−^ control only showed a trend in reduction relative to baseline and to a lesser extent compared to *Ldlr* ^−/−^/*Mmp14* ^SMC‐CKO^ mice (Figures 7F and S17A). Similarly, *en face* ORO staining of the aorta showed a substantial reduction in plaque area in *Ldlr* ^−/−^/*Mmp14* ^SMC‐CKO^ mice, while plaques in *Ldlr* ^−/−^ control mice did not differ from those in baseline (Figure 7G). On the other hand, collagen content within the plaques markedly increased with regression on chow diet in both *Ldlr* ^−/−^ control and *Ldlr* ^−/−^/*Mmp14* ^SMC‐CKO^ groups; however, no notable difference was seen between the two groups (Figures 7H and S17B). Nevertheless, these data indicate that inhibition of SMC MMP14 accelerates the regression of preexisting lesions.

## 4 DISCUSSION

**FIGURE 8:**
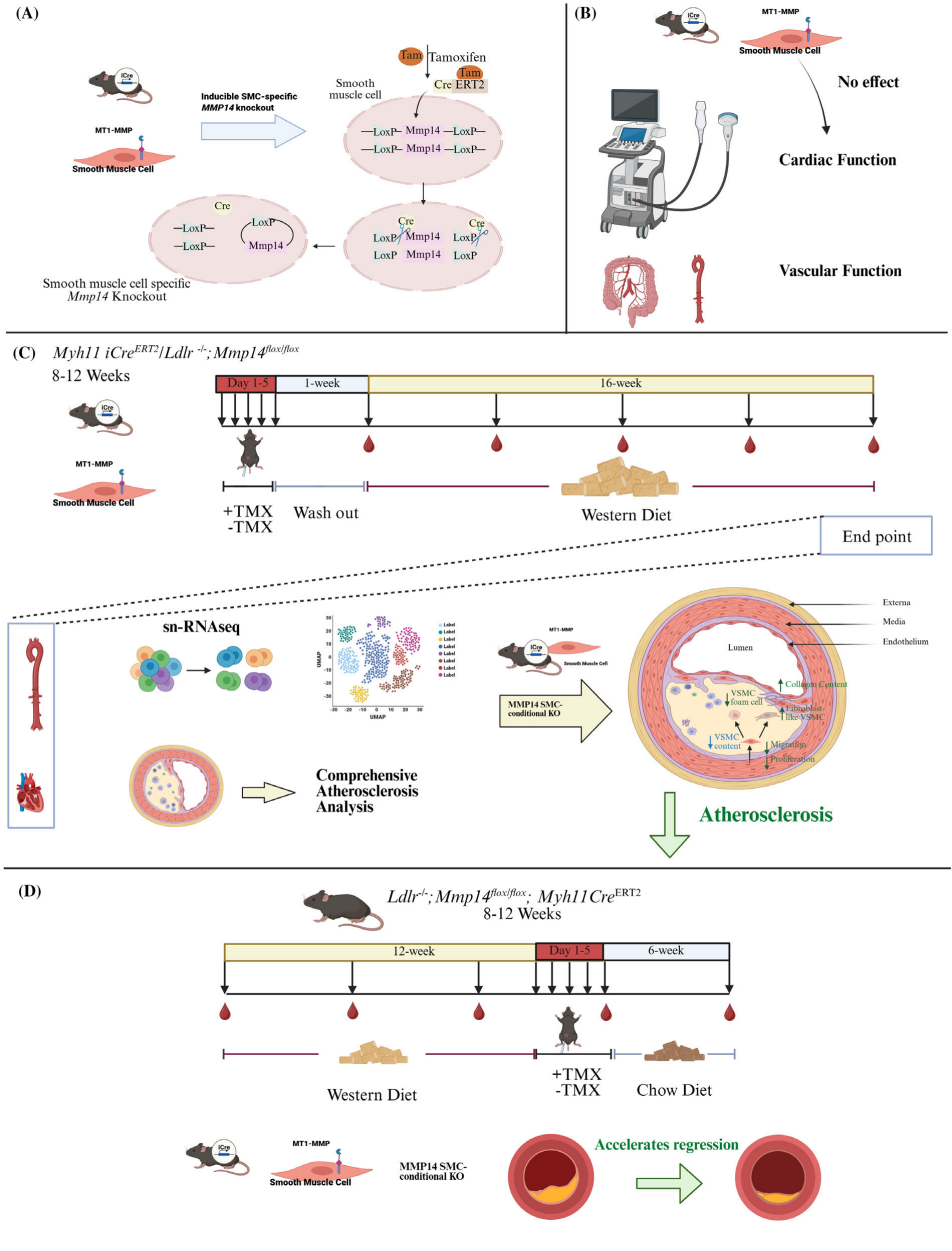
Inducible conditional SMC‐specific MMP14 knockout in mice. (A) Mechanism of inducible conditional SMC‐specific MMP14 knockout in mice (*Mmp14* ^SMC‐CKO^). (B) *Mmp14* ^SMC‐CKO^ does not affect basic cardiac or vascular function in mice. (C) *Mmp14* ^SMC‐CKO^ in *Ldlr^−/−^* mice reduces atherosclerosis progression after 16‐week WD. (D) *Mmp14* ^SMC‐CKO^ promotes atherosclerosis regression in *Ldlr^−/−^* mice.

In this study, we demonstrated that MMP14 deficiency in SMCs of adult mice alleviates atherosclerosis and promotes its regression without affecting plasma lipid levels or basic cardiovascular and vascular functions (Figure 8). Interestingly, a previous study reported that constitutive knockout of SMC MMP14 using smooth muscle 22α (SM22α)‐Cre mice increased atherosclerosis in *Apoe* ^−/−^ mice.^22^ In this model, MMP14 was deficient from the embryonic stage due to early SM22α promoter activity, which begins around embryonic Day 9.5 (E9.5).^41^ Given MMP14's essential role in development and its expression in embryonic arterial SMCs as early as E12.5,^42^, ^43^, ^44^ these models may have influenced vascular development. Indeed, the spontaneous formation of abdominal aneurysms was observed in these SMC *Mmp14* knockout mice,^22^ which could potentially confound the role of MMP14 in atherosclerosis. In addition, transgenic expression of mutant MMP14 (Y573D), which retains the catalytic activity, from the embryogenic stage also exacerbated atherosclerosis.^45^ Therefore, both MMP14 deficiency and overexpression during embryogenesis worsen atherosclerosis. Notably, MMP14 is prominently expressed within SMCs in the prospective arterial tunica media of large arteries in mice from early embryonic stages,^42^, ^43^, ^44^ and the protein is critical for development. Therefore, it is possible that artery development is affected in these mice, complexing the effects on atherosclerosis.

In contrast, we developed a conditional knockout of *Mmp14* in SMCs of adult mice using *Myh11*‐iCre/ERT2 mice. In these mice, Cre expression is controlled by a tamoxifen‐inducible *Myh11* promoter. The *Myh11*‐iCre/ERT2 mice show tamoxifen‐induced Cre activity specifically in SMCs and have been extensively utilized to generate inducible SMC knockout mice.^10^, ^25^, ^34^, ^35^, ^36^ We observed that conditional knockout of SMC MMP14 in adult male mice did not significantly affect basic vascular or cardiac function. Furthermore, we did not observe spontaneous formation of aneurysm when we induced the conditional SMC‐*Mmp14* knockout in adult mice. These findings indicate that the timing of MMP14 knockout in SMCs is a crucial contributor to the pathophysiological role of SMC‐MMP14 in atherosclerosis development.

In addition, SM22α‐Cre has been shown to be expressed in various other cell types, including adipocytes, platelets, myeloid cells, and cardiac fibroblasts, while Myh11‐Cre is believed to exhibit the most SMC‐specific expression of Cre.^46^, ^47^, ^48^ Moreover, *Apoe* ^−/−^ and *Ldlr* ^−/−^ mice were used in the two studies, respectively. While both models are commonly applied in atherosclerosis research, the underlying mechanisms differ. Lack of LDLR increases circulating LDL‐C levels and promotes atherosclerosis, while *Apoe* ^−/−^ mice exhibit impaired clearance of ApoE‐containing lipoprotein particles (such as chylomicron and VLDL remnants), promoting atherosclerosis.^49^, ^50^ APOE also functions in an anti‐atherogenic manner independent of lipoproteins, such as inflammation.^51^, ^52^, ^53^ Collectively, these differences may explain the discrepancies observed between the two studies.

sn‐RNA seq data showed that knockout of SMC MMP14 increased the subpopulation of fibroblast‐like SMCs while reducing the foam cell‐like subpopulation, suggesting a role for MMP14 in the phenotype switching of SMCs in atherosclerosis. Similar to our results, Lehti *et al*. reported that the absence of MMP14 increases the expression of contractile proteins in cultured SMCs.^40^ LRP1 is abundantly expressed in SMCs,^39^, ^54^ and the absence of LRP1 in SMCs promotes SMC proliferation and atherosclerosis development.^39^ In this study, we observed increased levels of LRP1 in MMP14‐deficient cells. Of note, PDGF‐BB promotes the proliferation and migration of vascular SMCs, processes that accelerate atherosclerosis.^55^, ^56^, ^57^ It has been reported that deletion of SMC‐LRP1 increases PDGFRβ levels and promotes its signalling.^58^ Importantly, we observed that MMP14 deficiency also reduced PDGFRβ levels, attenuated PDGF signalling, and decreased SMC proliferation and migration. However, LRP1 knockdown did not rescue the reduced proliferation or migration in MMP14‐deficient SMCs, indicating that increased LRP1 expression is unlikely to be the cause of the effect of MMP14 deficiency on SMC phenotypic change. It also suggests that the reduction in SMC dedifferentiation induced by MMP14 deficiency may be achieved through a different mechanism.

MMP14 also cleaves collagen, including collagen type I, which is the most abundant collagen in the intima and atherosclerotic plaques.^59^ SMCs need to degrade the collagen barrier to migrate; moreover, collagen degradation within the atherosclerotic plaques can reduce plaque stability. We observed impaired collagenolytic activity in arterial explants from conditional SMC‐*Mmp14* knockout mice, indicating that MMP14 is a key metalloproteinase responsible for degrading proximal collagen around SMCs. Additionally, we showed that deficiency of MMP14 in SMCs leads to upregulated expression of genes related to ECM synthesis, including *Col1a1*, *Col1a2*, *Col3a1* and *Fn1*, indicating more ECM‐producing SMCs. MMP14 deficiency also increased the fibroblast‐like SMC cluster compared to the control group. These may explain the increased collagen content within plaques despite the reduction in SMC content in conditional SMC‐*Mmp14* knockout mice. The reduction in collagenolytic activity caused by MMP14 deficiency may also contribute to the diminished migration of SMCs and the subsequent reduction in SMCs within the plaques.

In conclusion, knockout of SMC MMP14 at adulthood not only reduces the development of atherosclerosis but, more importantly, accelerates the regression of existing atherosclerosis, a more clinically relevant event. In addition, we have demonstrated that the absence of hepatic MMP14 increases LDLR levels in the liver and reduces plasma LDL cholesterol levels, alleviating atherosclerosis.^18^ While the deletion of MMP14 in bone marrow‐derived macrophages increases plaque stability.^20^ Therefore, inhibition of MMP14 can target several mechanisms, lowering LDL‐C levels, reducing SMC migration and proliferation, and increasing plaque stability, all of which mitigate atherosclerosis. Of note, *Mmp14* ^−/−^ mice exhibit multiple severe abnormalities and typically die 3–4 weeks after birth.^16^, ^17^ Conditional global knockout of MMP14 in adult mice results in severe arthritis.^60^ Therefore, systematic inhibition of MMP14 may cause unwanted side effects. Conversely, conditional MMP14‐deficient mice specifically in the epidermis, monocytes/macrophages, or hepatocytes appear indistinguishable from their wild‐type counterparts.^18^, ^61^, ^62^ Silencing SMC MMP14 in adult mice also does not significantly affect basic cardiovascular functions. MMP14 is a transmembrane protein, offering significant advantages over soluble MMPs for cell‐type‐specific targeting. N‐acetyl galactosamine (GalNAc) conjugation has been successfully used for hepatic‐specific delivery.^63^, ^64^ Recent studies have reported highly selective targeting of VSMCs using specific nanoparticles or technologies.^65^, ^66^, ^67^ For example, Wu et al. developed a cell penetrating peptides‐conjugated liposome‐polycation‐DNA complex (CLPD) that effectively targets VSMCs. CLPD loaded with siRNA against ribonucleotide reductase M2 can inhibit SMC migration and proliferation.^66^ Additionally, Chin et al. developed micelles that selectively delivered miR145 to vascular SMCs, mitigating atherosclerosis in *Apoe* ^−/−^ mice.^68^ Therefore, cell‐type‐specific targeting of MMP14 may represent a promising therapeutic approach for patients who do not effectively respond to current treatments.

### 4.1 Limitations

When we initiated this project, the commonly used mouse models for generating inducible SMC‐specific knockout were Myh11‐iCreERT2 mice, in which the transgene is inserted on the Y chromosome. This limitation confined our studies to male mice. Recently, *Myh11‐CreERT2‐RAD* mice, featuring a chromosome 2 insertion of the transgene, have been developed.^34^ These mice exhibit tamoxifen‐inducible, SMC‐specific Cre activity in both sexes. Further research is warranted to explore the effect of SMC MMP14 on atherosclerosis in female mice.

*Translational outlook*: These findings pave the way for new therapeutic strategies targeting SMC dedifferentiation in atherosclerosis management.

## AUTHOR CONTRIBUTIONS

D.W.Z. designed the experiments, analyzed data, supervised and directed this project, and wrote the manuscript. S.J. performed the bulk of the experiments, collected and analyzed data. S.J. and D.W.Z. wrote the first draft of the manuscript. F.S., A.C., A.Y., D.H., R.P., H.M.G., G.G., P.A., M.E.G., and X.D.X. performed experiments, collected and analyzed data. Y.W. provided guidance on the use of human biospecimens and input on data interpretation. G.F., Y.W., J.M.S., S.T.D., and X.D.X. provided technical support and guidance, helpful discussions and comments.

## CONFLICT OF INTEREST STATEMENT

The authors declare no conflicts of interest.

## Supporting information



Supporting Information

## Data Availability

The data that support the findings of this study are available from the corresponding author upon reasonable request.
