# Supplementary material for: Conditional knockout of membrane‐type I matrix metalloproteinase in smooth muscle cells of adult mice alleviates atherosclerosis without affecting basic cardiovascular function
Source: Clin Transl Med. 2026 Jul 7;16(7):e70739. doi: 10.1002/ctm2.70739 (PMC13341647; doi:10.1002/ctm2.70739)
Supplement: Supplementary file 1 — Supporting Information [file CTM2-16-e70739-s001.docx]

**Supplementary Data**

**Table S1-2**

**Figure S1-17**

**Conditional knockout of membrane-type I matrix metalloproteinase in smooth muscle cells of adult mice alleviates atherosclerosis without affecting basic cardiovascular function**

Suha Jarad^a^, Hong-mei Gu^b^, Daniel Huang^a^, Peter Amadi^b^, Govind Gill^b^, Floor Spaans^c,d^, Aakar Chatha^e,f^, Ala Yousef^g^, Murilo E. Graton^c,d^, Raj Patel^a^, John M. Seubert^g,h^, Ying Wang^e,f^, Gordon Francis^f,i^, Xiao-dan Xia^j^*, Sandra T. Davidge^c,d,k^* and Da-wei Zhang^a,b^*

^a^ Department of Biochemistry, Group on Molecular and Cell Biology of Lipids, University of Alberta. ^b^ Department of Pediatrics, Group on Molecular and Cell Biology of Lipids, University of Alberta. ^c^ Departments of Obstetrics/ Gynecology, University of Alberta, ^d^ Women and Children’s Health Research Institute, University of Alberta. ^e^ Department of Pathology and Laboratory Medicine, University of British Columbia. ^f^ Centre for Heart Lung Innovation, University of British Columbia. ^g^ Faculty of Pharmacy and Pharmaceutical Sciences, University of Alberta. ^h^ Department of Pharmacology, Faculty of Medicine and Dentistry, University of Alberta. ^i^ Department of Medicine, University of British Columbia. ^j^ Affiliated Qingyuan Hospital, Guangzhou Medical University, Qingyuan People's Hospital. ^k^ Department of Physiology, University of Alberta.

**Running title:** MMP14 and atherosclerosis

* **Corresponding authors:**

Xia-dan Xia (xiaxiaodan1979@gzhmu.edu.cn), Affiliated Qingyuan Hospital, Guangzhou Medical University, Qingyuan People's Hospital, Qingyuan, Guangdong 511518, China.

Sandra Davidge ([sdavidge@ualberta.ca](mailto:sdavidge@ualberta.ca)), Departments of Obstetrics/ Gynecology and Physiology, 232 HMRC, University of Alberta, Edmonton, Alberta, CANADA T6G 2S2

Da-wei Zhang ([dzhang@ualberta.ca](mailto:dzhang@ualberta.ca)), Departments of Pediatrics and Biochemistry, Group on Molecular and Cell Biology of Lipids, 303 HMRC, University of Alberta, Edmonton, Alberta, CANADA T6G 2S2

**Table S1. Human Atherosclerotic Lesion Samples Patients**' **Characteristics.**

| Sample ID | Origin | Sex | Age (year) | Pathology diagnosis | Clinical diagnosis |
| --- | --- | --- | --- | --- | --- |
| 11498 H20 | 11498 | M | 52 | Fibroatheroma | Ischemic cardiomyopathy |
| 11969 H20 | 11969 | F | 66 | PIT | Ischemic cardiomyopathy |
| 12190 H20 | 12190 | M | 59 | PIT | Ischemic cardiomyopathy |
| 12462 H21 | 12462 | M | 63 | Fibroatheroma | Ischemic cardiomyopathy |
| 12464 H20 | 12464 | M | 53 | PIT | Ischemic cardiomyopathy |
| 12667 H21 | 12667 | M | 62 | Fibroatheroma | Ischemic cardiomyopathy |
| 12819 H19 | 12819 | M | 63 | Fibroatheroma | Ischemic cardiomyopathy |
| 13621 H19 | 13621 | F | 56 | PIT | Ischemic cardiomyopathy |
| 15137 H20 | 15137 | M | 68 | PIT | Ischemic cardiomyopathy |
| 15243 H19 | 15243 | M | 63 | Fibroatheroma | Ischemic cardiomyopathy |
| 17704 H19 | 17704 | F | 56 | PIT | Ischemic cardiomyopathy |
| 17704 H22 | 17704 | F | 56 | Fibroatheroma | Ischemic cardiomyopathy |
| 19405 H20 | 19405 | M | 57 | PIT | Ischemic cardiomyopathy, dilated cardiomyopathy |
| 19405 H21 | 19405 | M | 57 | Fibroatheroma | Ischemic cardiomyopathy, dilated cardiomyopathy |

**Table S2. Primer sequence.**

| **Name** | **Forward** | **Reverse** |
| --- | --- | --- |
| **qRT-PCR** | | |
| Mouse *Gapdh* | AACAGCAACTCCCACTCTTC | CCTGTTGCTGTAGCCGTATT |
| Human *GAPDH* | GGTGTGAACCATGAGAAGTATGA | GAGTCCTTCCACGATACCAAAG |
| Mouse *Mmp14* | TGACAGGCAAGGCTGATTT | CCTTGATCTCAGTCCCAAACTTA |
| Mouse *Acta2* | CCATCATGCGTCTGGACTT | GGCAGTAGTCACGAAGGAATAG |
| Mouse *Cnn1* | TTGAGAGAAGGCAGGAACATC | GTACCCAGTTTGGGATCATAGAG |
| Mouse *Tagln* | CTAATGGCTTTGGGCAGTTTG | CTGTCTGTGAAGTCCCTCTTATG |
| Mouse *Col1a1* | GCTTGAAGACCTATGTGGGTATAA | GGTGGAGAAAGGAGCAGAAA |
| Mouse *Col1a2* | CCAGAGTGGAACAGCGATTAC | GATGCAGGTTTCACCAGTAGAG |
| Mouse *Col3a1* | GGATGGGTTCTGCTCTCATATT | CCTCTGCTCTGGCCTTAAATAG |
| Mouse *Fn1* | TCCTGTCTACCTCACAGACTAC | GTCTACTCCACCGAACAACAA |
| **siRNA** | | |
| Negative control | AUUAGUGUGCGAUGUACCCAGGAAC | GUUCCUGGGUACAUCGCACACUAAUAU |
| Human MT1-MMP | CGCCGACUAAGCAGAAGAAAGAUCA | UGAUCUUUCUUCUGCUUAGUCGGCGAA |
| Mouse *Lrp1* | GAUACCAUUGAGGUGUCC | AACUCCACAGGUUCGAAU |
| **Genotyping** | | |
| *Mmp14*flox | F1: CCTACCATGGGCATAACCTG  F2: AGGGTGCAGACAGATGGAAG | AGGGTGCAGACAGATGGAAG |
| *Myh11-Cre* | TGACCCCATCTCTTCACTCC | AGTCCCTCACATCCTCAGGTT |
| *Ldlr-/-* | TATGCATCCCCAGTCTTTGG | ATA GAT TCG CCC TTG TGT CC |

**Figures**

**
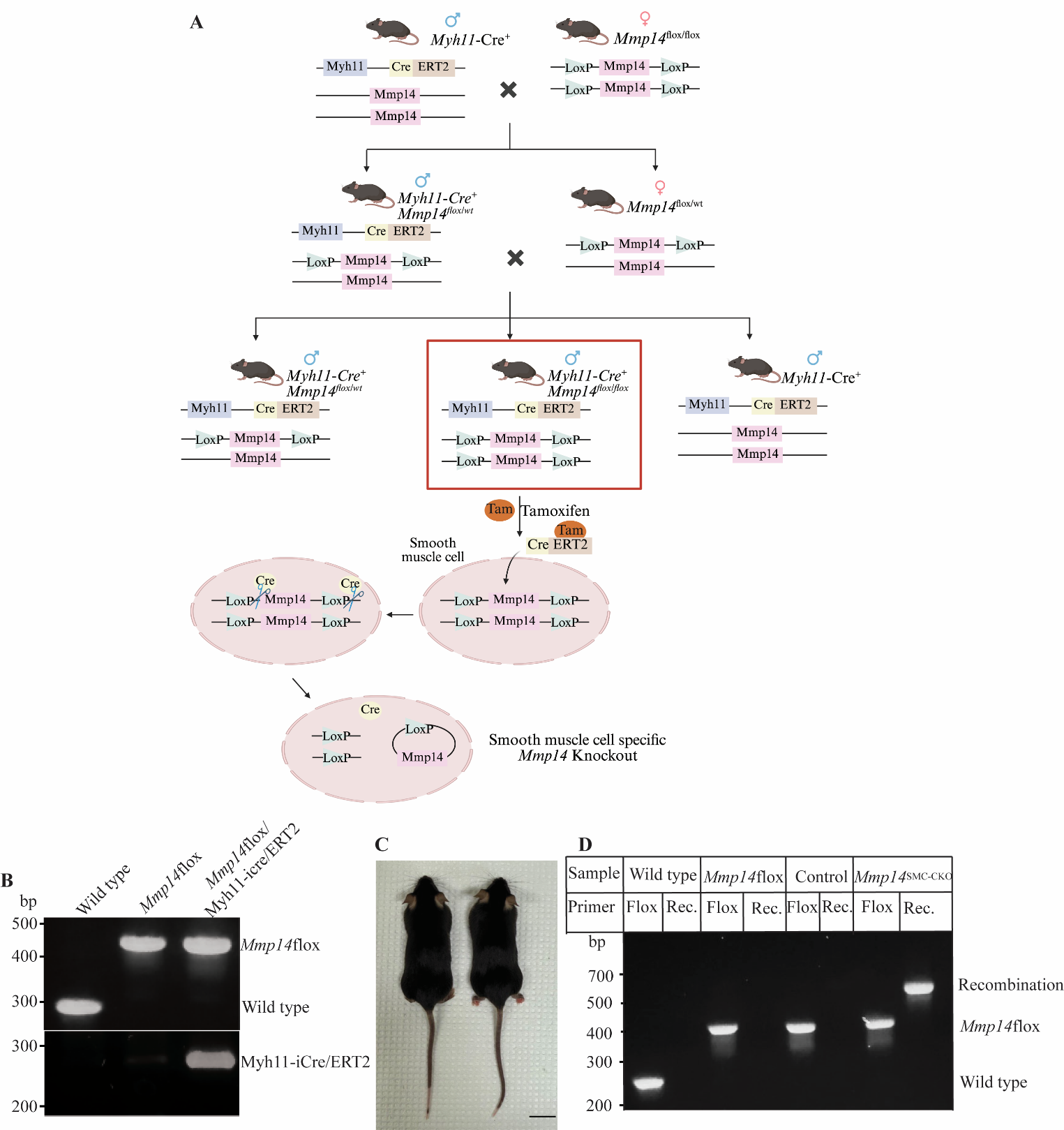
**

**Figure S1: Inducible SMC-specific *Mmp14* knockout in mice. A,** Schematic illustration for the generation of SMC-specific MMP14 knockout in mice, male *Myh11*-Cre/ERT2 mice were bred with female *Mmp14*^flox^ mice to generate *Mmp14*^flox^/*Myh11*-Cre/ERT2 mice. **B,** Genotyping. **C,** Representative picture of control and *Mmp14*^SMC-CKO^ mice (scale bar, 1cm). **D,** Genotyping of wild type, Mmp14 flox and recombination bands to confirm recombination.

**
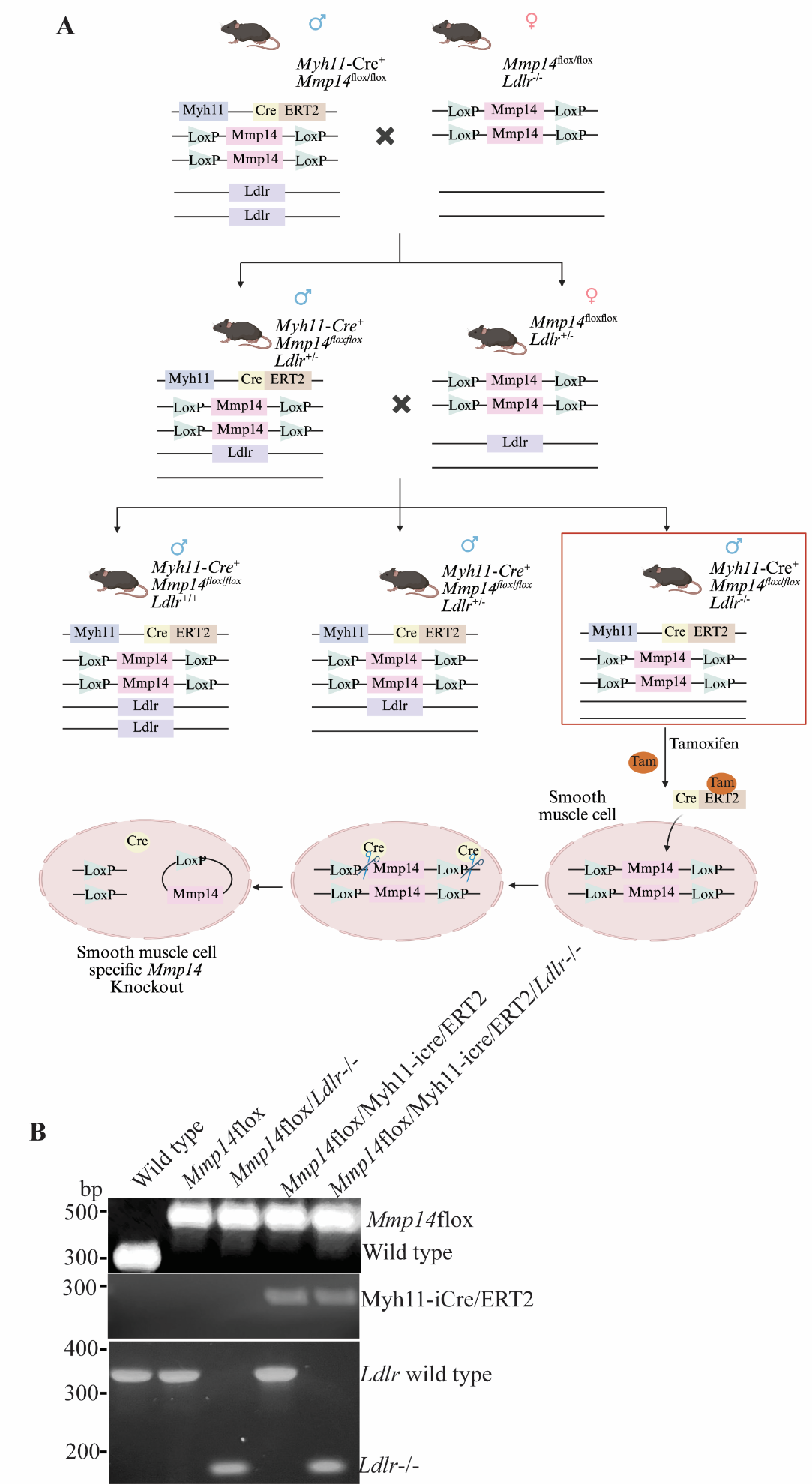
**

**Figure S2: Inducible SMC-specific *Mmp14* knockout in *Ldlr^-/-^* mice. A,** Schematic illustration for the generation of SMC-specific MMP14 knockout in *Ldlr^-/-^* mice. Male *Myh11*-Cre^+^/*Mmp14*^flox^ mice were bred with female *Mmp14*^flox^/*Ldlr^-/^*^-^ mice to generate *Myh11*-Cre^+^/*Mmp14*^flox^/*Ldlr^-/-^* mice. **B,** Genotyping showing the presence of the floxed *Mmp14* gene and the absence of the *Ldlr* gene in *Mmp14*^flox^/*Ldlr^-/^*^-^ mice, as well as the presence of the floxed *Mmp14* gene, *Myh11*-Cre gene, and the absence of the *Ldlr* gene in *Ldlr^-/-^* /*Mmp14*^flox^/ *Myh11*- Cre^/ERT2^ mice.


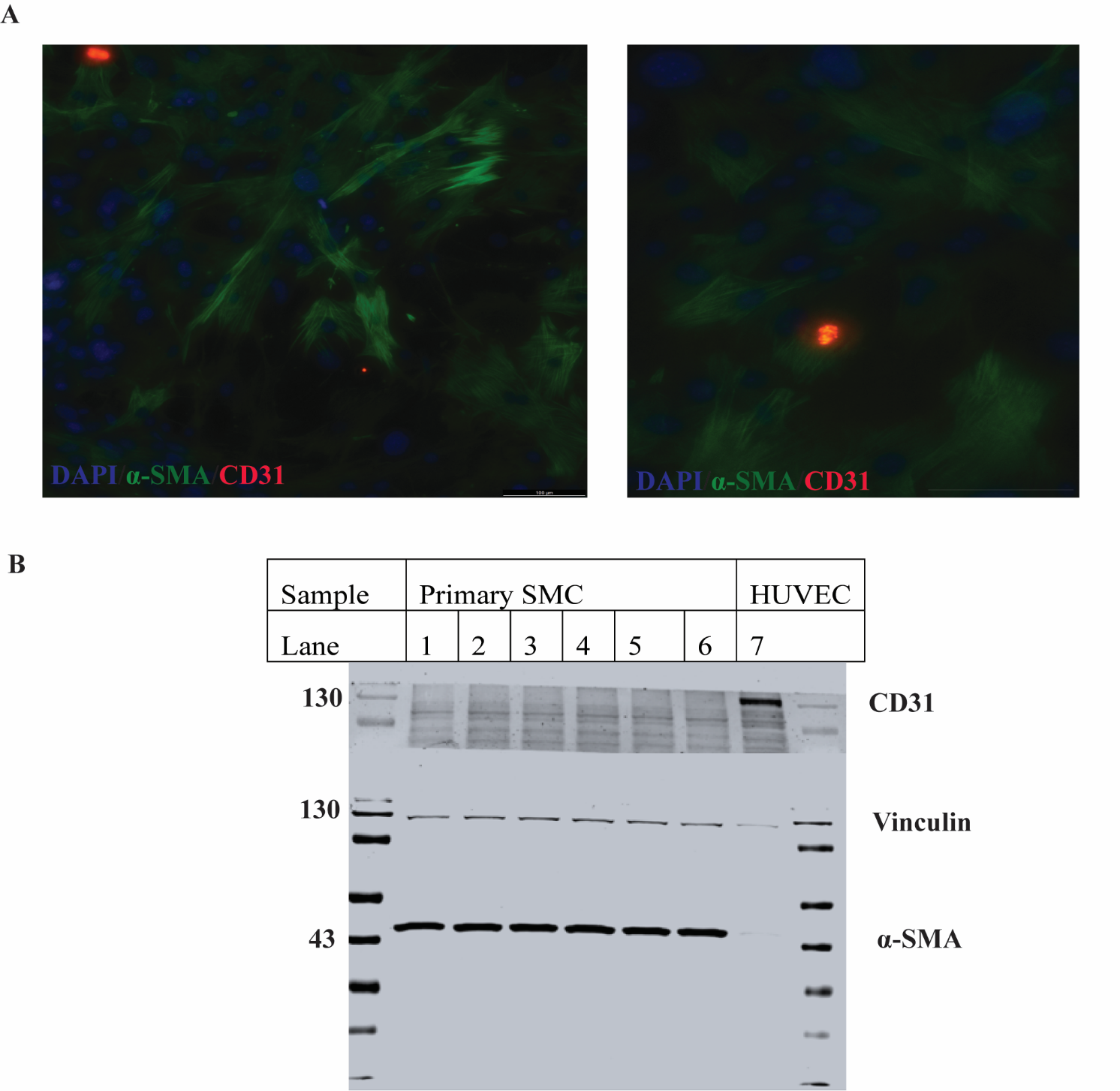


**Figure S3: Identity of isolated primary smooth muscle cells. A,** Immunofluorescence staining of DAPI (blue), α-SMA (green), and CD31 (red) of isolated primary smooth muscle cells (scale bar=100µm) **B,** CD31 and α-SMA protein expression in lysates of primary SMCs (lanes 1-6) and HUVEC (lane 7), Vinculin is used as loading control on the same blot.


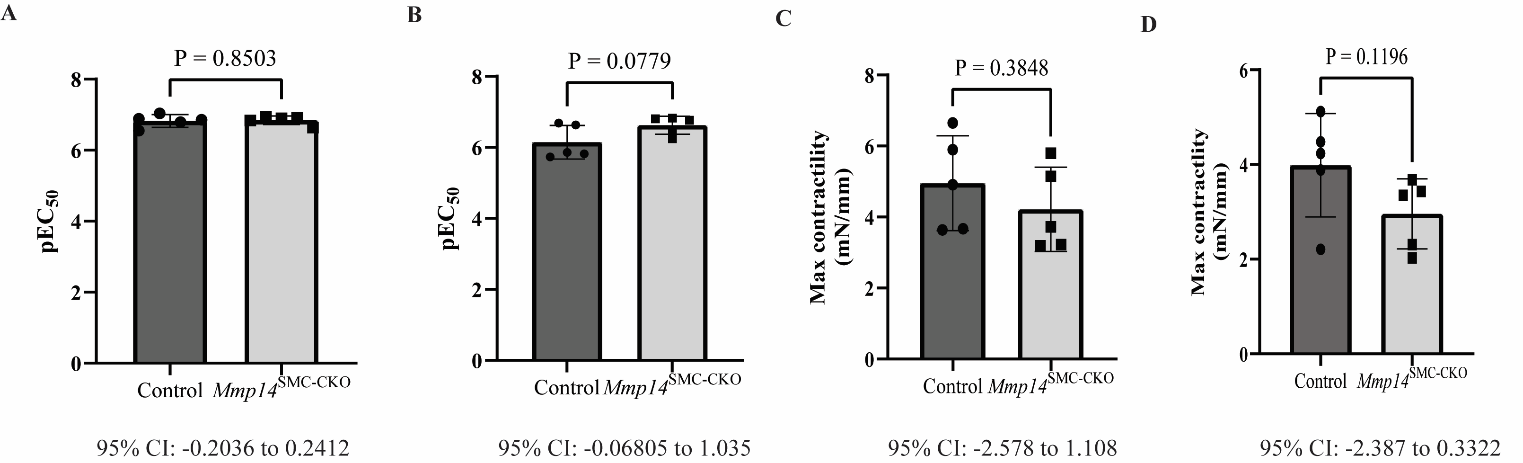


**Figure S4: Effects of SMC-specific *Mmp14* knockout in adult mice on vascular function. A and B,** assessment of pEC50 in the thoracic (A) and abdominal aorta (B) of control and *Mmp14*^SMC-CKO^ mice (n=5/group). **C and D,** The maximum contractility (Emax) of the thoracic (C) and abdominal aorta (D) of control and *Mmp14*^SMC-CKO^ mice (n=5/group). n refers to biological replicates. Data are represented as mean ± S.D. *P*-value was calculated by unpaired two-tailed Student's t-test. *P*-value <0.05 is considered significant.

**
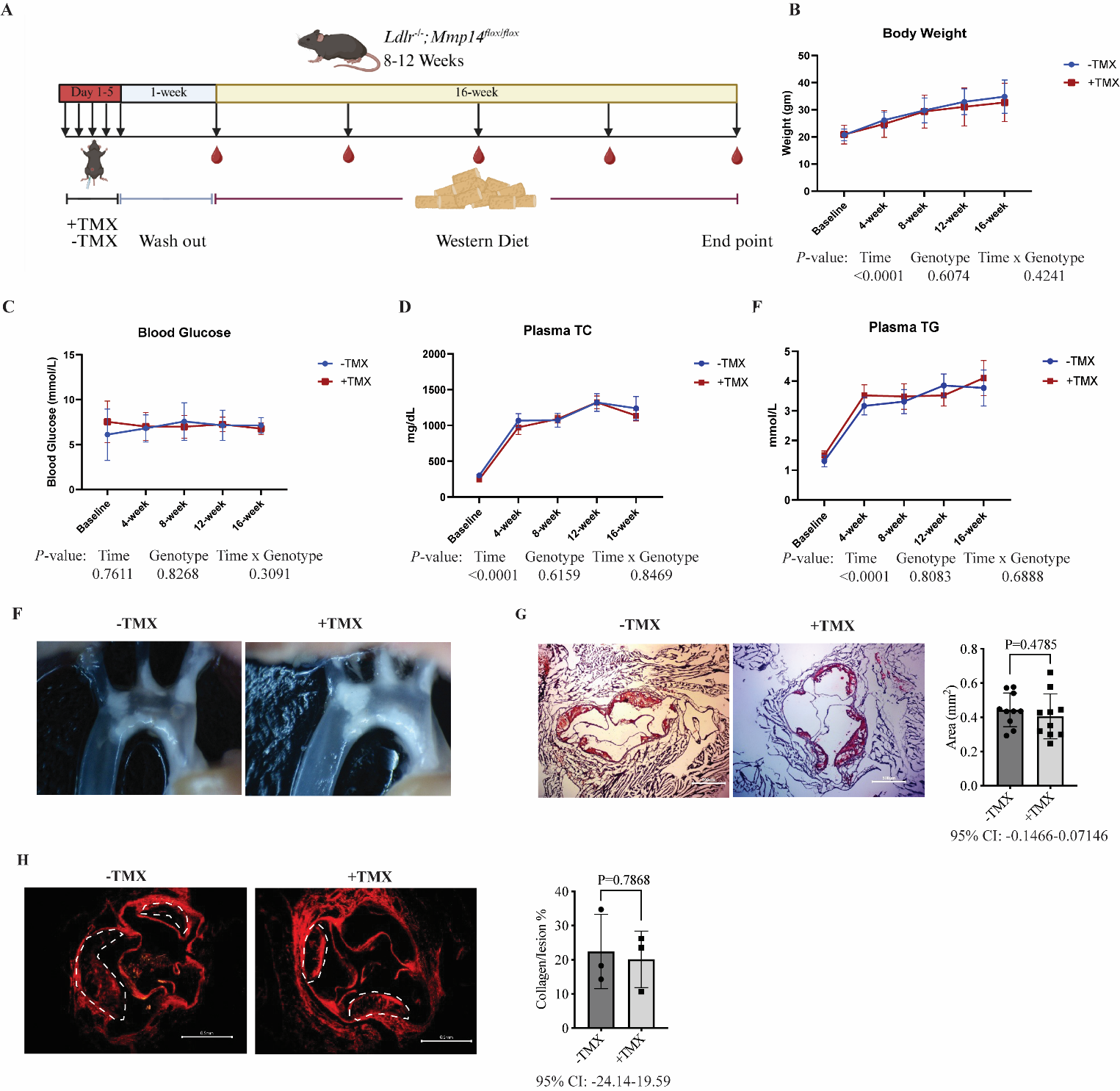
Figure S5: Effect of tamoxifen on atherosclerosis progression. A,** Graphical illustration of the study design, 8-12 week male *Ldlr^-/^*^-^/*Mmp14*^flox^ mice were administered tamoxifen (+TMX) or olive oil (-TMX) for 5 consecutive days, followed by 1-week washout, then mice were changed to a Western Diet (WD) for 16 weeks. Blood samples were collected every 4 weeks. **B and C,** Body weight (B) and blood glucose (C) of -TMX and +TMX mice (n=10 mice per group). **D and E,** Plasma total cholesterol (TC, D) and plasma triglycerides (TG, E) of -TMX and +TMX mice (n=10 mice per group). **F,** Representative pictures of the aortic arch of -TMX and +TMX mice at the endpoint. **G,** Representative pictures and quantification of oil red O-stained aortic sinus of -TMX and +TMX mice at endpoint (n=10 mice per group, scale bar= 0.5mm). **H,** Representative pictures and quantification of picrosirius red-stained aortic sinus of -TMX and +TMX mice at the end point (n=3 mice per group, scale bar= 0.5mm). Data are represented as mean ± S.D. *P*-value was calculated by linear mixed effects model with mouse ID included as a random effect (REML) in panels B-E, by unpaired two-tailed Student's t-test in panels G and H. Dashed lines indicate plaque areas in quantification. n indicates biological replicates. *P*-value <0.05 is considered significant.

**
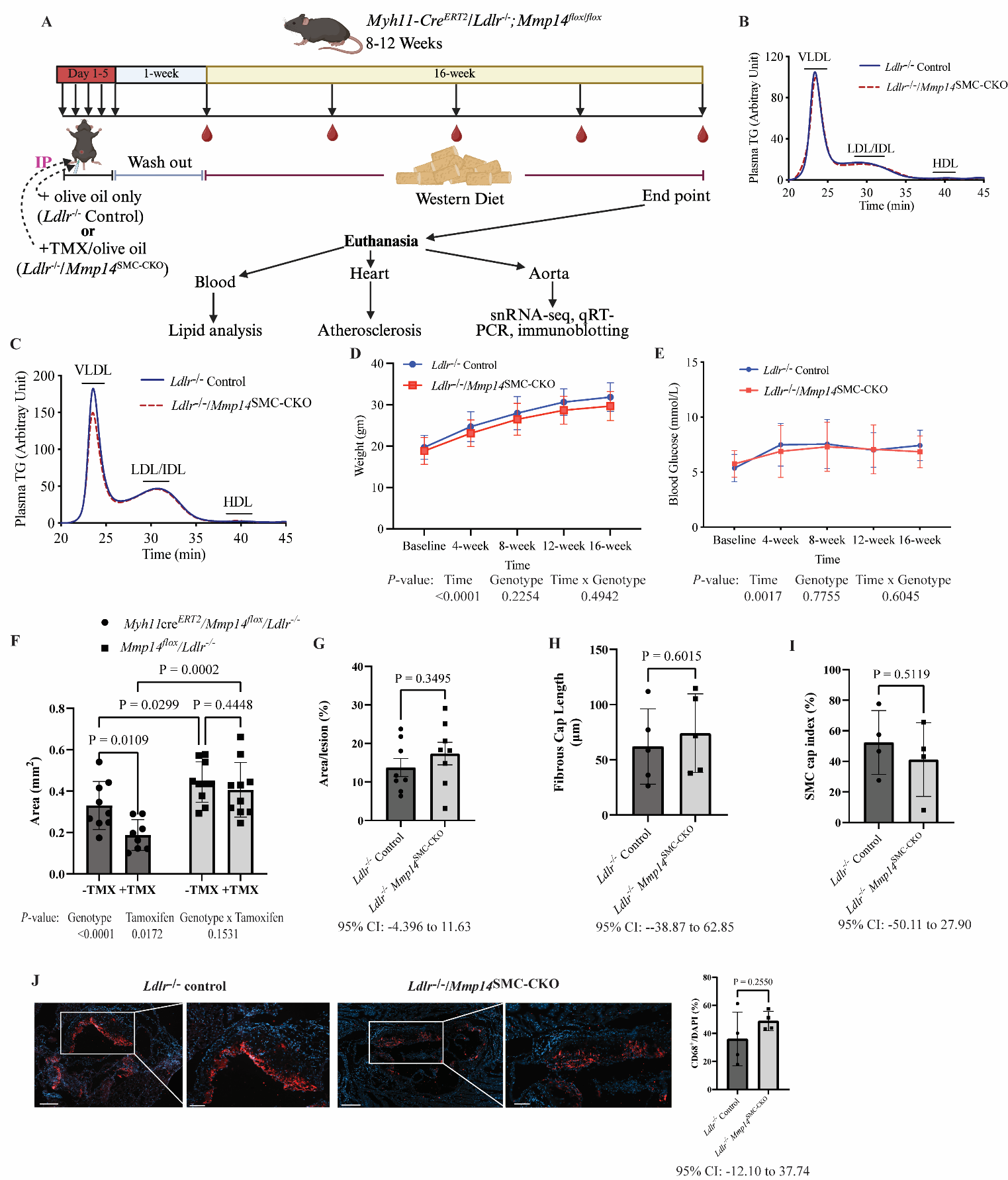
Figure S6: Effects of SMC-specific *Mmp14* knockout on *Ldlr*^-/-^ control and *Ldlr*^-/-^/*Mmp14*^SMC-CKO^ mice. A,** Graphical illustration of the study design, 8-12 week *Ldlr^-/^*^-^/*Mmp14*^flox^/*Myh11*-Cre mice (male) were administered tamoxifen (+TMX/olive oil, *Ldlr*^-/-^/*Mmp14*^SMC-CKO^) or olive oil only (*Ldlr*^-/-^ control) for 5 consecutive days, followed by 1-week washout. Mice were then changed to a Western diet (WD) for 16 weeks. Blood samples were collected and body weight was measured every 4 weeks. Tissues and blood were collected at the endpoint for further analysis as indicated. **B and C,** FPLC of fasting plasma TG levels of control and *Ldlr*^-/-^/*Mmp14*^SMC-CKO^ mice at baseline (B) and the endpoint (C) (n=6 /group). **D and E,** Body weight (D) and blood glucose (E) of control and *Ldlr*^-/-^/*Mmp14*^SMC-CKO^ mice (n=12mice per group). **F,** Quantification of oil red O-stained aortic sinus of all groups in *Myh11cre*^ERT2^/*Mmp14*^flox^/*Ldlr*^-/-^ and *Mmp14*^flox^/*Ldlr*^-/-^ mice at the endpoint (n=8-10/group). **G,** Quantification of necrotic core area in oil red O-stained aortic sinus of *Ldlr*^-/-^ control and *Ldlr*^-/-^/*Mmp14*^SMC-CKO^ mice (n=8/group). **H,** Quantification of fibrous cap length of picrosirius red-stained aortic sinus of *Ldlr*^-/-^ control and *Ldlr*^-/-^/*Mmp14*^SMC-CKO^ mice (n=6/group). **I,** smooth muscle cell cap index in aortic sinus of *Ldlr*^-/-^ control and *Ldlr*^-/-^/*Mmp14*^SMC-CKO^ mice (n=4/group). **J,** Immunofluorescence staining and quantification of DAPI (blue) and CD68 (red) in the aortic sinus of *Ldlr*^-/-^ control and *Ldlr*^-/-^/*Mmp14*^SMC-CKO^ mice (n=4/group) at the endpoint (scale bar=200µm, inset picture scale bar=100µm). n indicates biological replicates. *P*-value was calculated by linear mixed effects model with mouse ID included as a random effect (REML) in panels D and E, two-way ANOVA followed by Tukey post hoc analysis in panel F, or by unpaired two-tailed Student's t-test in panels G-J. *P*-value <0.05 is considered significant.

**
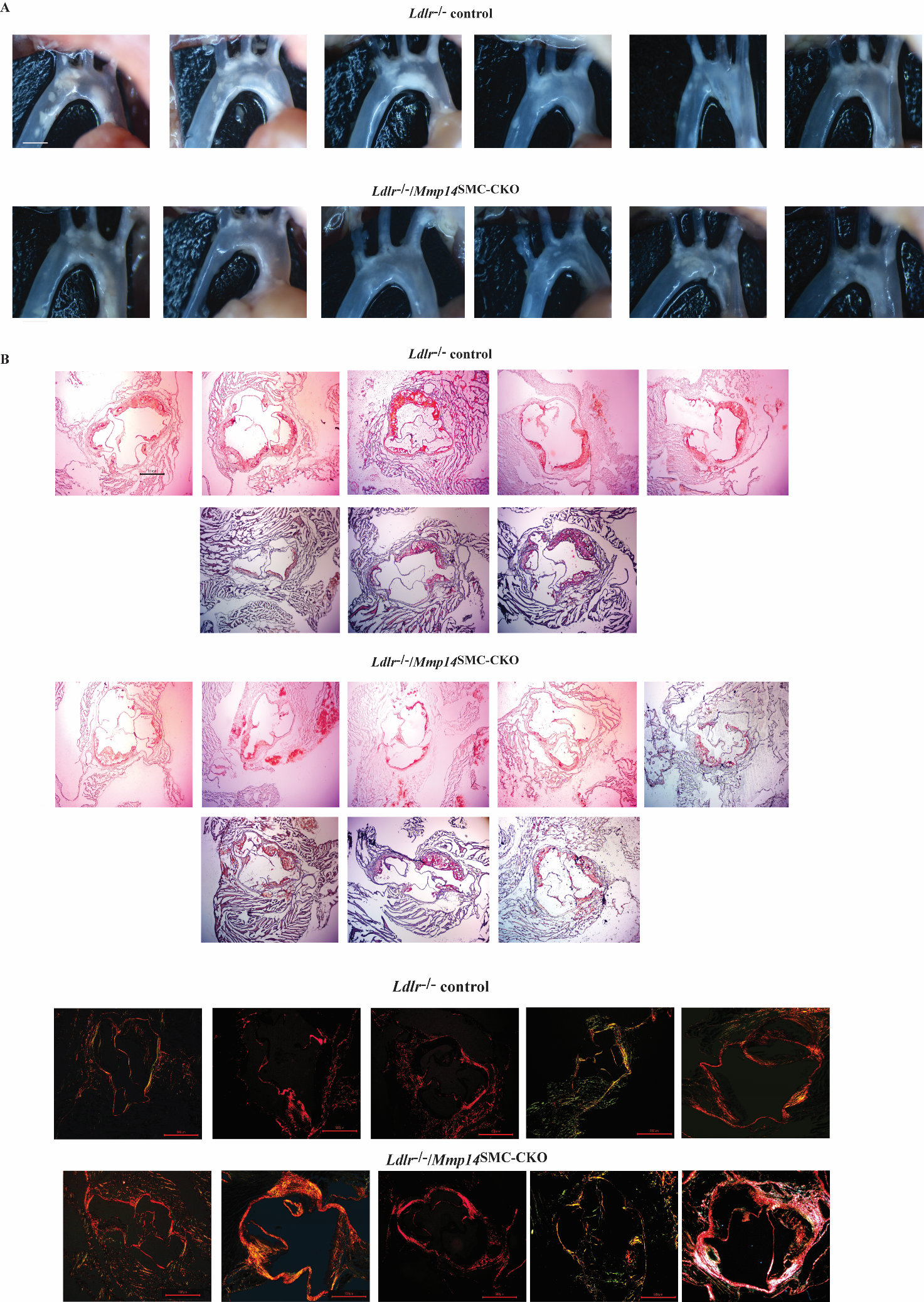
**

**Figure S7: Atherosclerosis progression in *Ldlr*^-/-^ control and *Ldlr*^-/-^/*Mmp14*^SMC-CKO^ mice**. **A-C** Pictures of aortic arch (A), oil-red-O stained aortic sinus (B) and picrosirius red-stained aortic sinus (C) of *Ldlr*^-/-^ control and *Ldlr*^-/-^/*Mmp14*^SMC-CKO^ mice (n=6-8/group).**
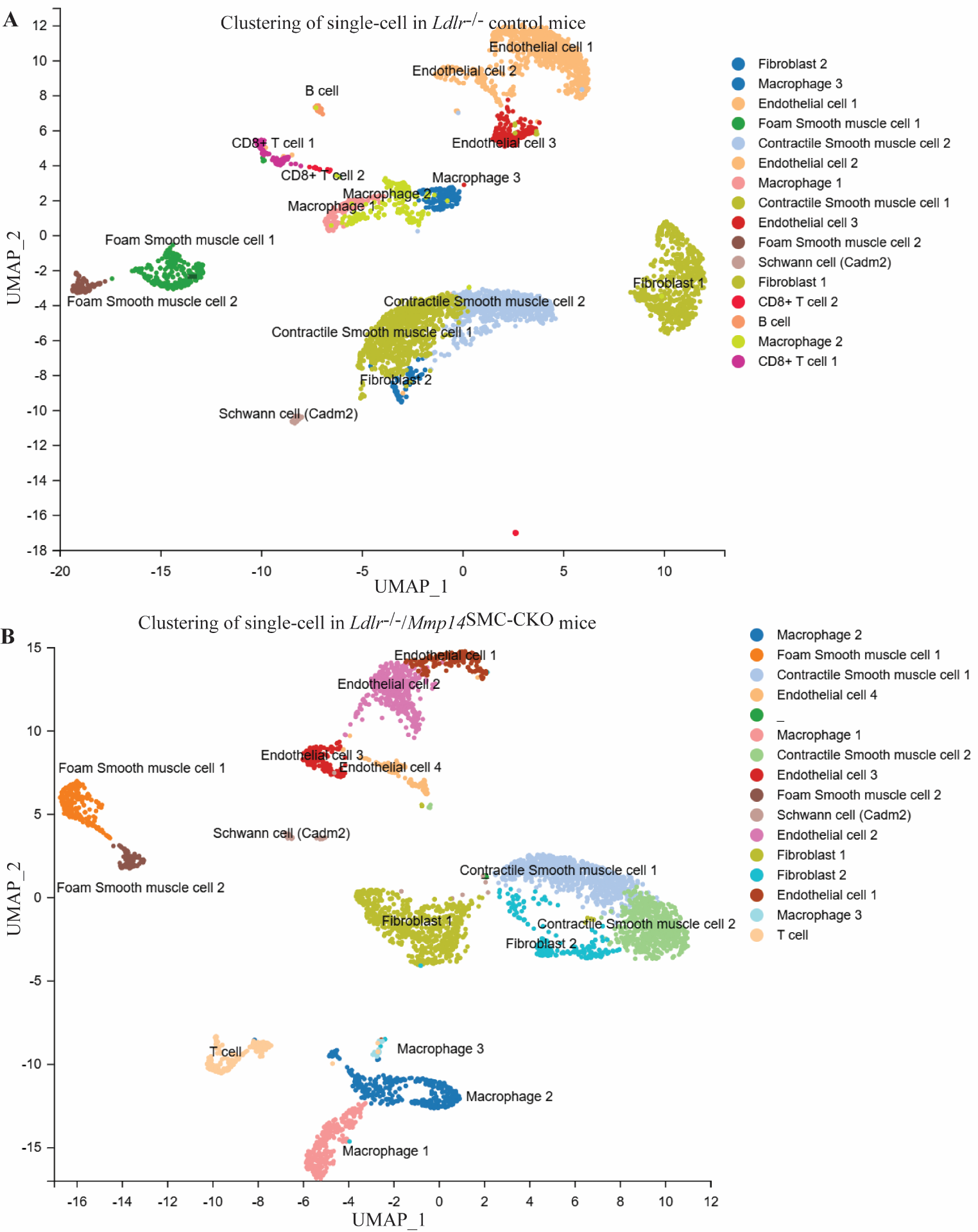
Figure S8: Single cell RNA seq analysis. A and B,** UMAP analysis showing cell groups from the control (*Ldlr^-/^*^-^/*Mmp14*^flox^/*Myh11*-Cre mice receiving olive oil) and *Ldlr*^-/-^/*^Mmp14^*^SMC-CKO^ mice (*Ldlr^-/^*^-^/*Mmp14*^flox^/*Myh11*-Cre mice receiving tamoxifen). Different cell clusters are indicated in different colors.

**
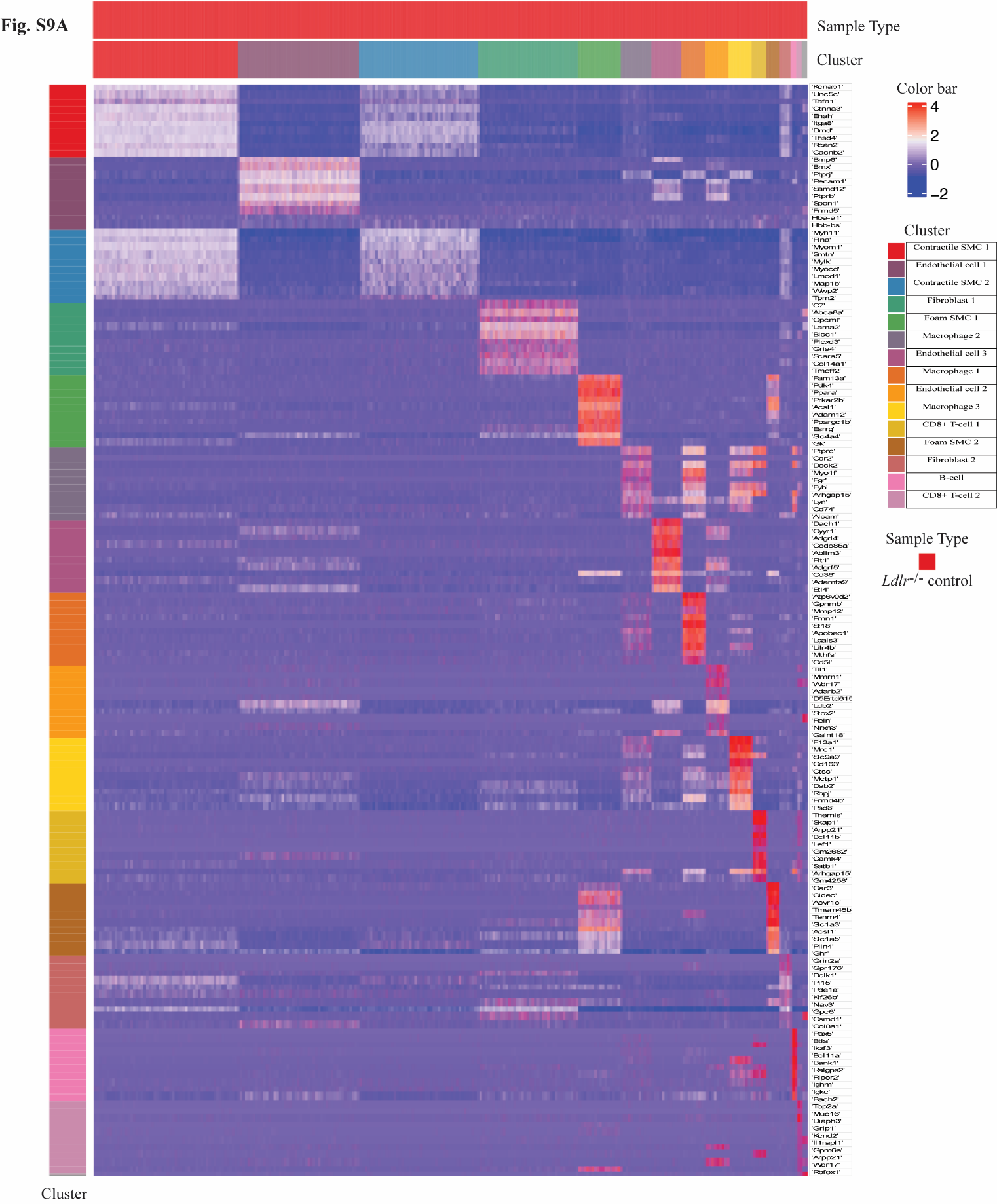

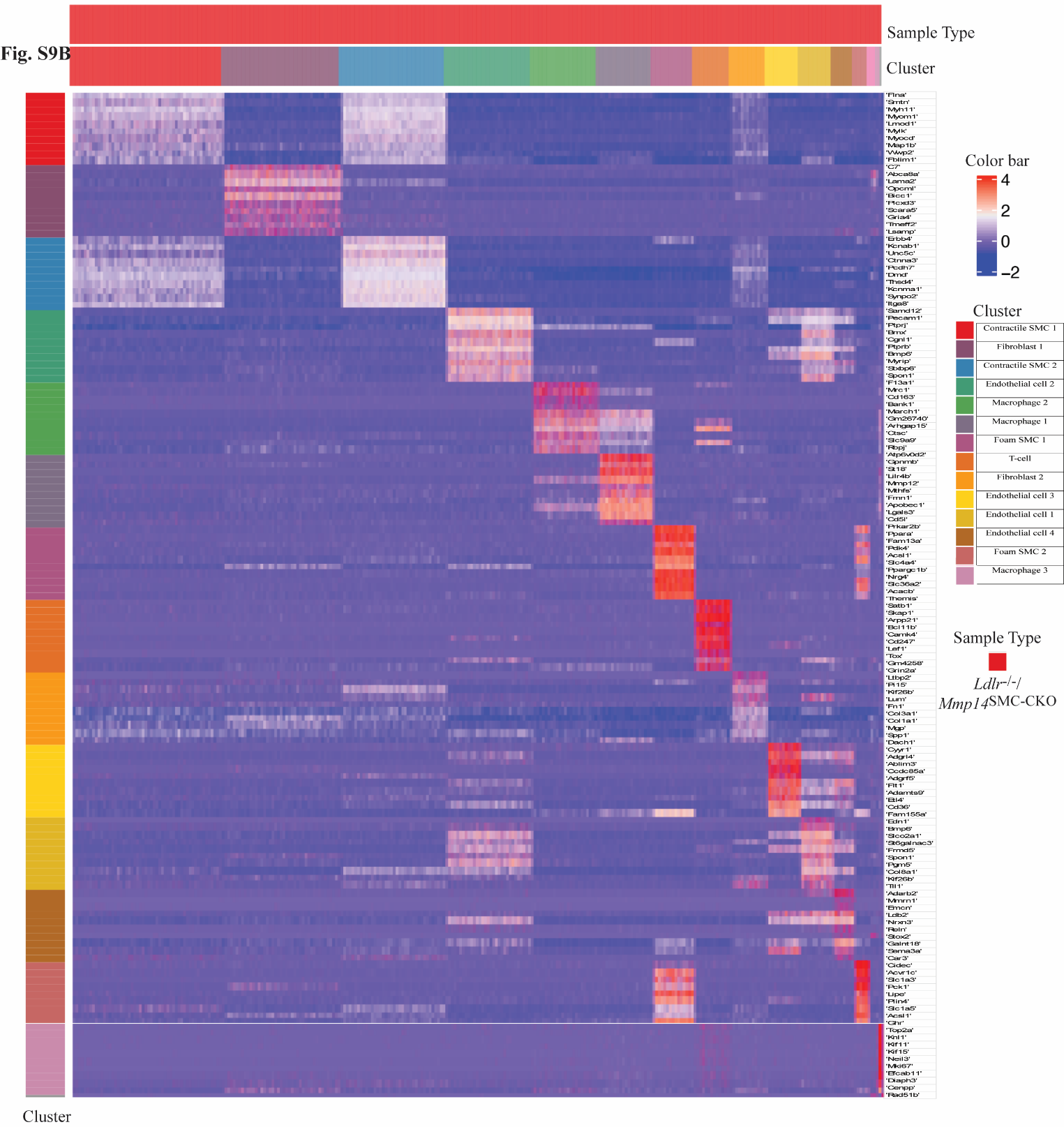
Figure S9: Single cell RNA seq analysis. A and B,** Heat map shows the expression of the top 10 genes in each cluster in *Ldlr*^-/-^ control (A) and *Ldlr*^-/-^/*^Mmp14^*^SMC-CKO^ mice (B).

**
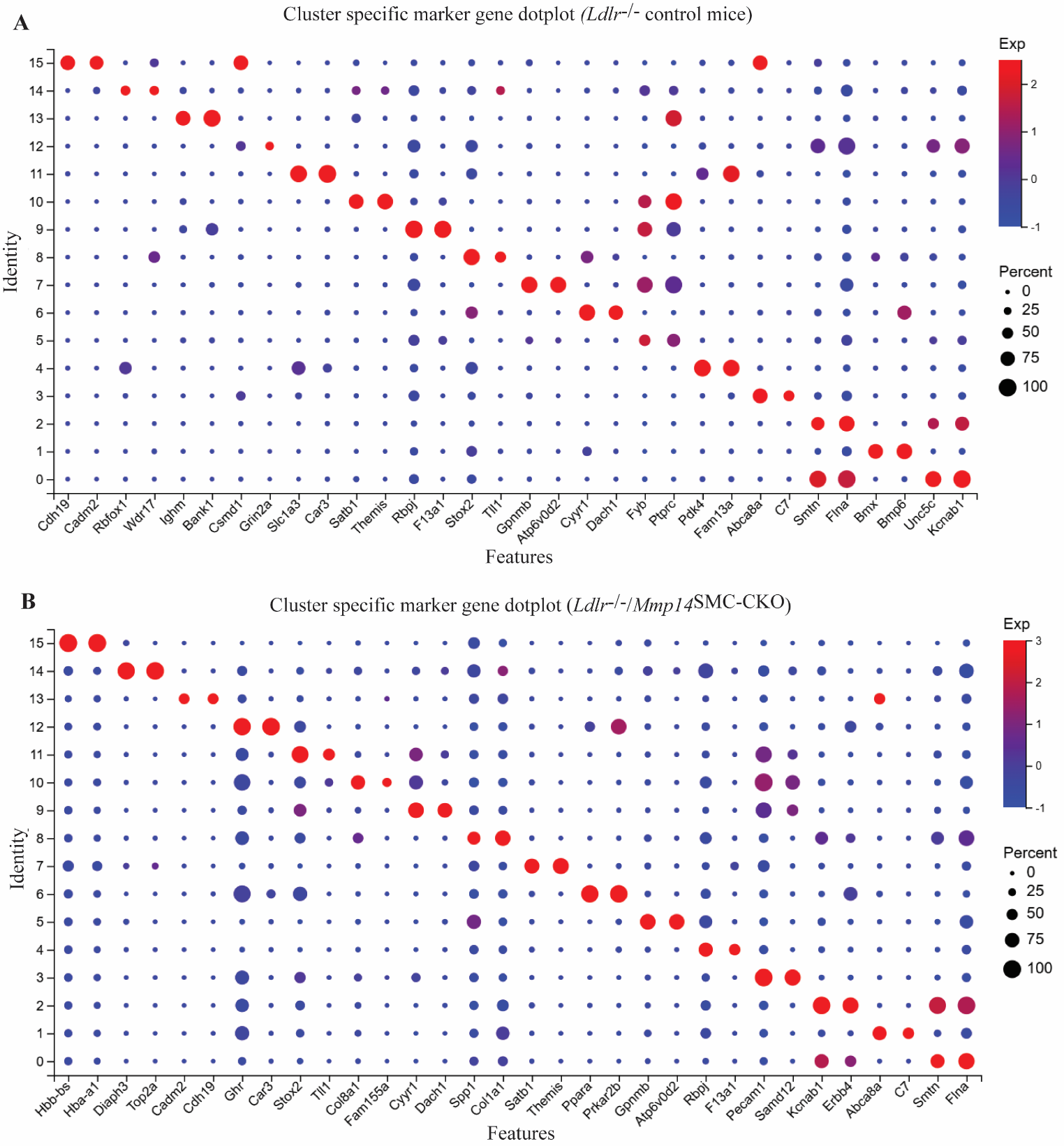
**

**Figure S10: Single cell RNA seq analysis. A and B,** Dot blot shows the expression of the top two genes expressed in each cluster in *Ldlr*^-/-^ control (A) and *Ldlr*^-/-^/*^Mmp14^*^SMC-CKO^ mice (B).

**
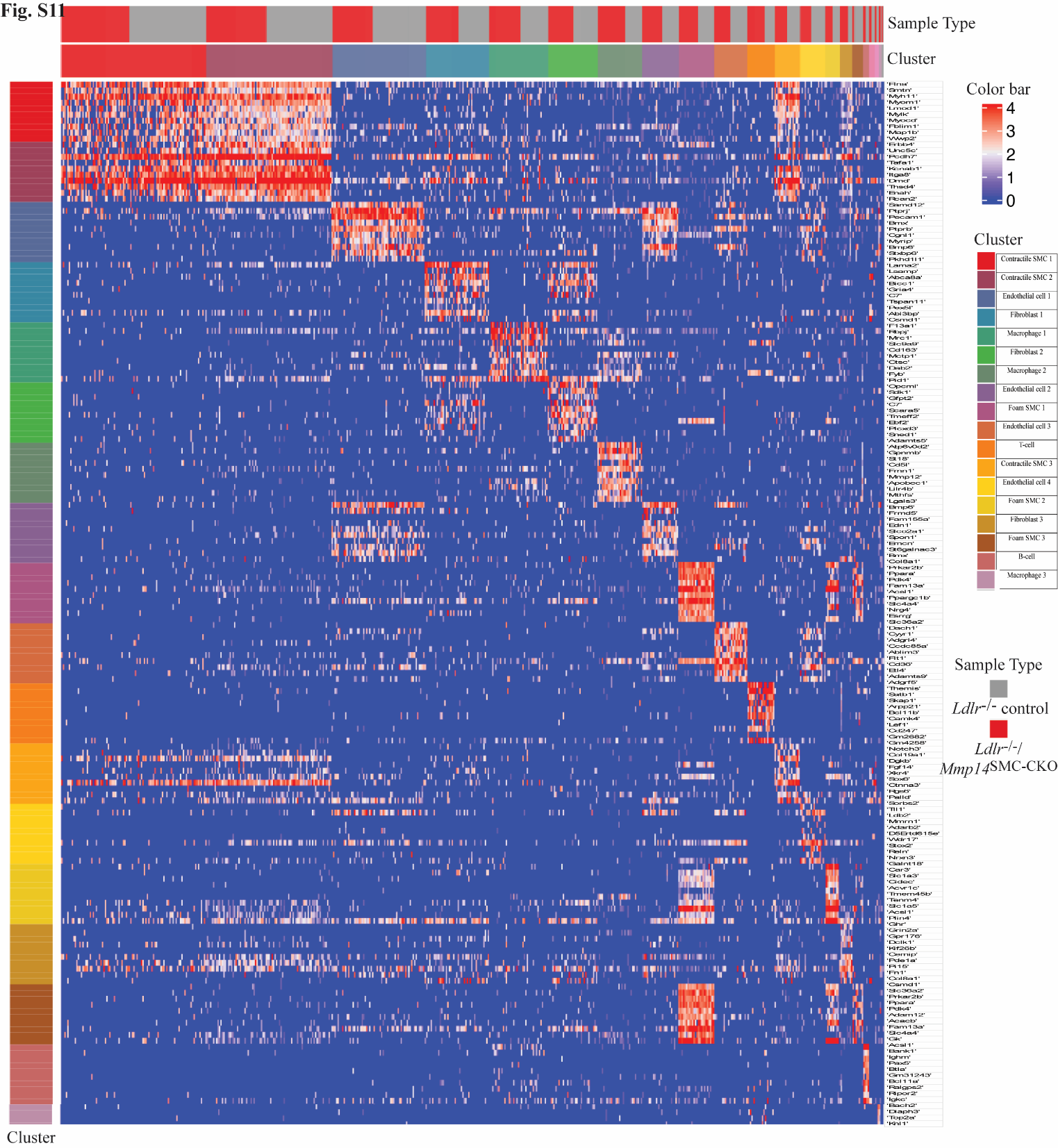
Figure S11: Single cell RNA seq analysis.** Heat map shows the expression of the top 10 genes in the combined library of both groups.

**
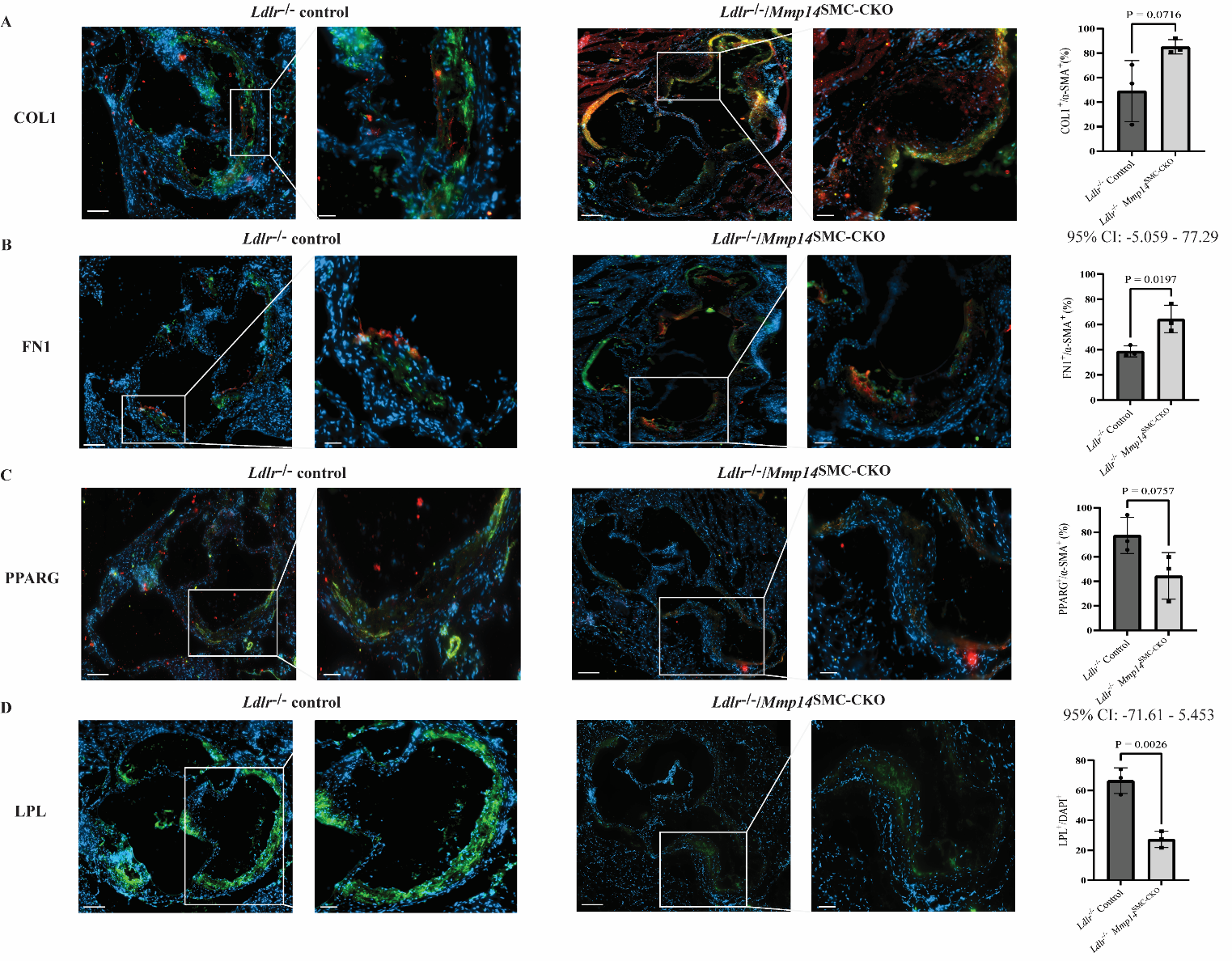
Figure S12: Expression of COL1, FN1, PPARG, and LPL within lesions of *Ldlr*^-/-^ control and *Ldlr*^-/-^/*Mmp14*^SMC-CKO^ mice. A-C,** Immunofluorescence staining of DAPI (blue), α-SMA (green), COL1A1 (red) **(A)**, FN1 (red) (B), PPARG (red) (C) in the aortic sinus of *Ldlr*^-/-^ control and *Ldlr*^-/-^/*Mmp14*^SMC-CKO^ mice (n=3/group) at the endpoint and quantification of cells expressing the indicated protein relative to α-SMA expressing cells. **D,** Immunofluorescence staining and quantification of DAPI (blue) and LPL (green) in the aortic sinus of *Ldlr*^-/-^ control and *Ldlr*^-/-^/*Mmp14*^SMC-CKO^ mice (n=3/group) at the endpoint (scale bar=100µm, inset picture scale bar=50µm). Data are represented as mean ± S.D. *P*-value was calculated by unpaired two-tailed Student's t-test. *P*-value <0.05 is considered statistically significant.

**
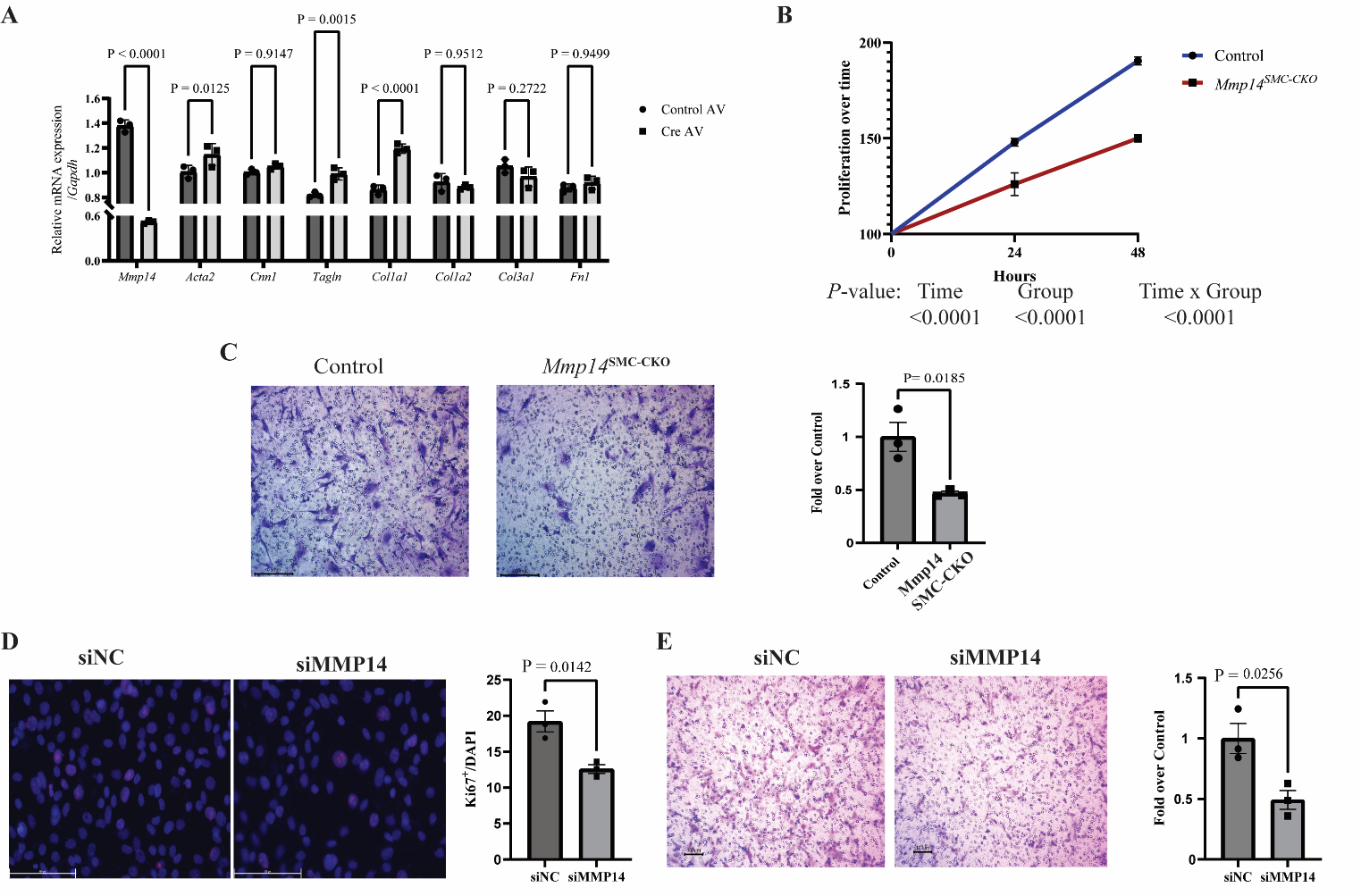
Figure S13: Effects of MMP14 deficiency on SMC proliferation and migration. A,** Relative mRNA levels of *Acta2*, *Cnn1*, *Tagln*, *Col1a1*, *Col1a2*, *Col3a1*, *Fn1*, and *Mmp14* to *Gapdh* in primary SMCs isolated from *Mmp14*^flox^ mice infected with control-AV or Cre-AV (n=3). **B,** Proliferation of primary SMCs isolated from control and *Mmp14*^SMC-CKO^ mice over 48 hours (n=9). **C,** Representative pictures and quantification for primary SMCs migrated through an uncoated transwell (n=3, scale bar=0.5mm). **D,** Representative pictures and quantification for HASMCs stained with DAPI (blue) and Ki67 (red) (n=3, scale bar= 100 µm). **E,** Representative pictures and quantification for HASMCs migrated through an uncoated transwell (n=3, scale bar=100 µm). n indicates biological replicates. Data are represented as mean ± S.D. *P*-value was calculated by two-way ANOVA followed by Tukey post hoc analysis in panel A and B, by unpaired two-tailed Student's t-test in panels C to E. *P*-value <0.05 is considered statistically significant.


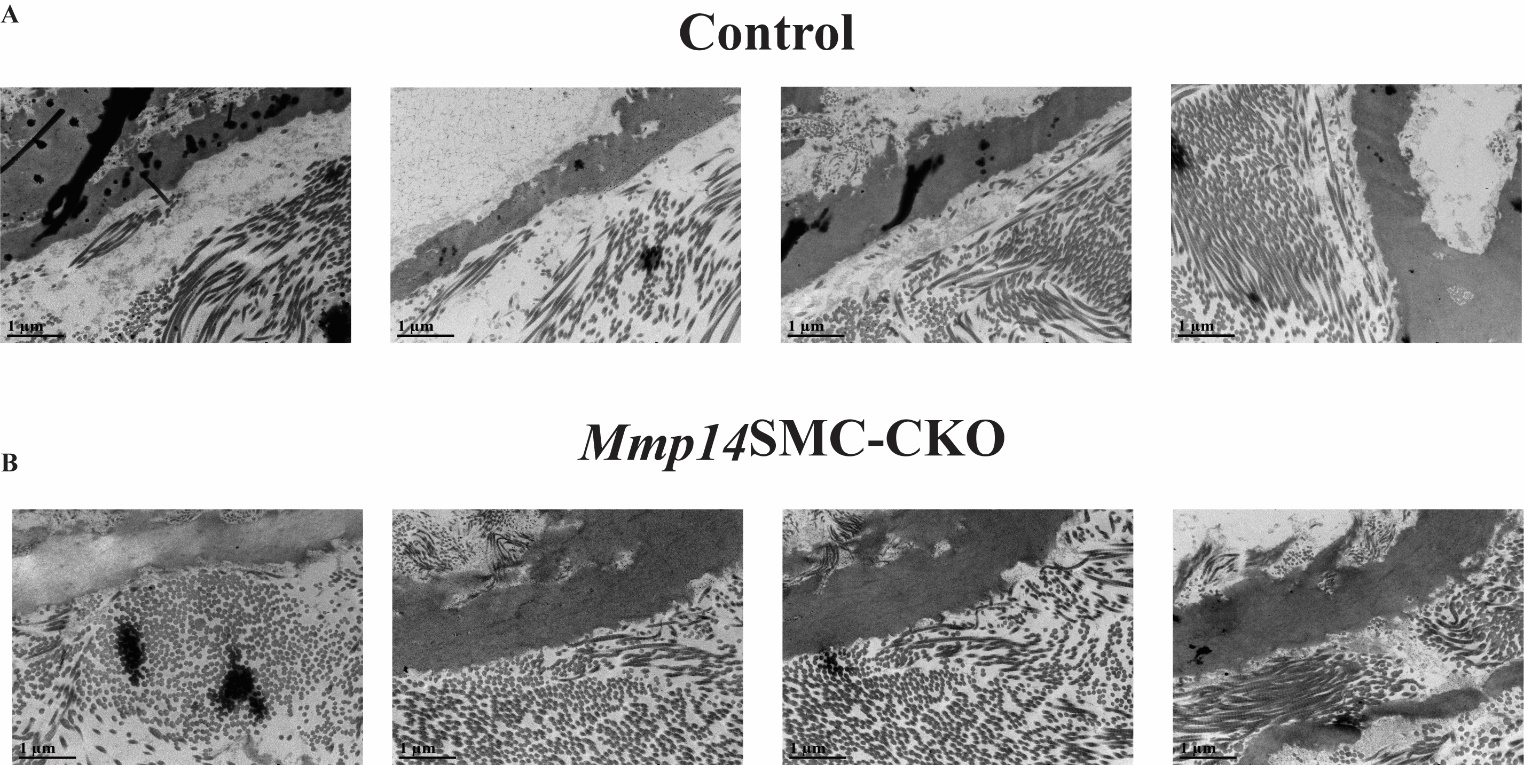


**Figure S14: Electron microscopy pictures of control explant (A) and *Mmp14*^SMC-CKO^ explant (B) cultured in type I collagen gel.** (scale bar=1 µm).

**
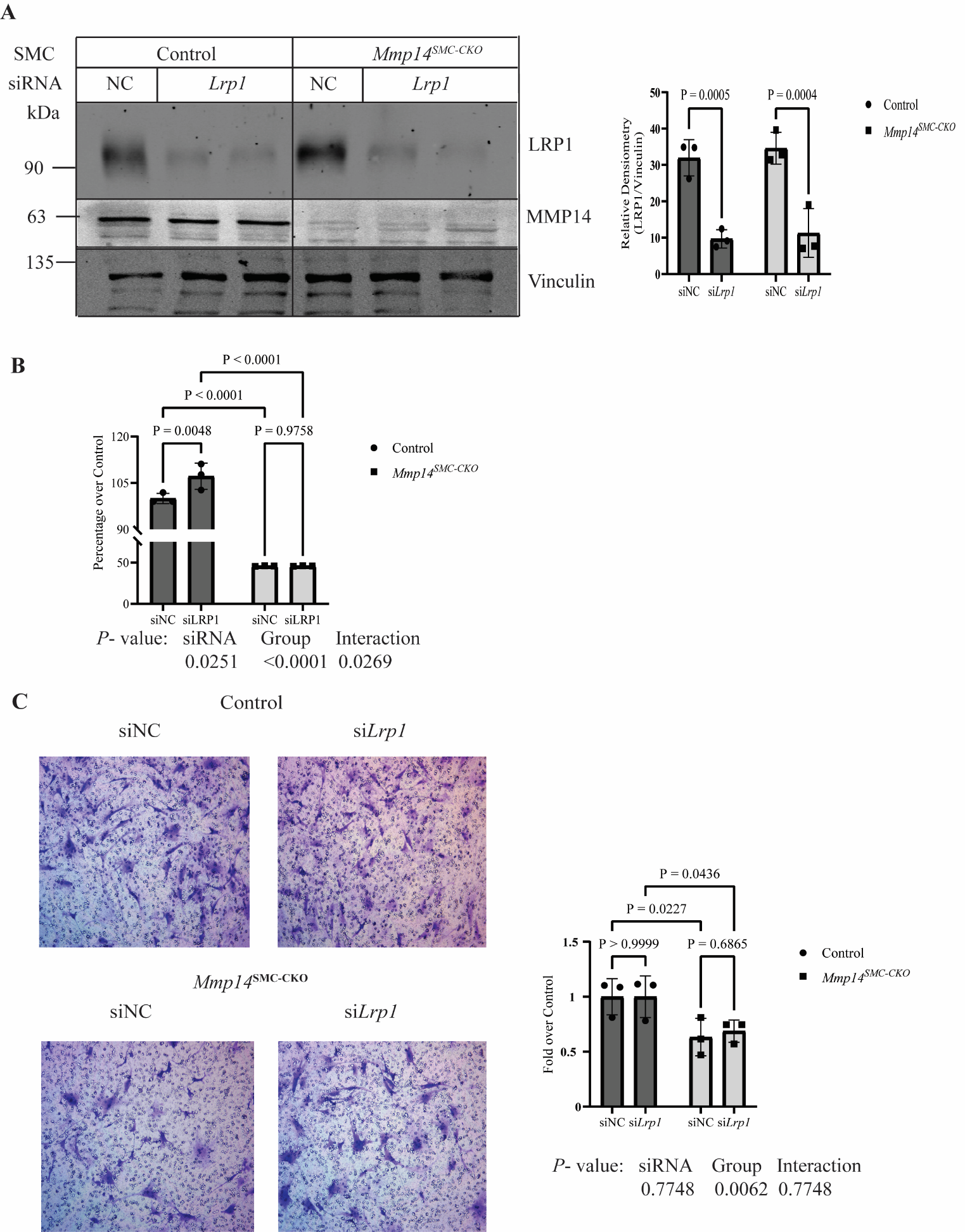
Figure S15: Effect of LRP1 knockdown on primary SMC proliferation and migration. A,** LRP1 protein expression and its quantification, which is normalized to Vinculin on the same blot in primary SMCs transfected with NC or *Lrp1* siRNA (n=3). **B,** Proliferation of primary SMCs isolated from control and *Mmp14*^SMC-CKO^ mice and transfected with NC or *Lrp1* siRNA (n=3). **C,** Representative pictures and quantification of control and si*Lrp1* primary SMCs migrated through uncoated transwell (n=3, scale bar=100µm). Data are represented as mean ± S.D. *P*-value was calculated by two-way ANOVA followed by Tukey post hoc analysis. *P*-value <0.05 is considered statistically significant.


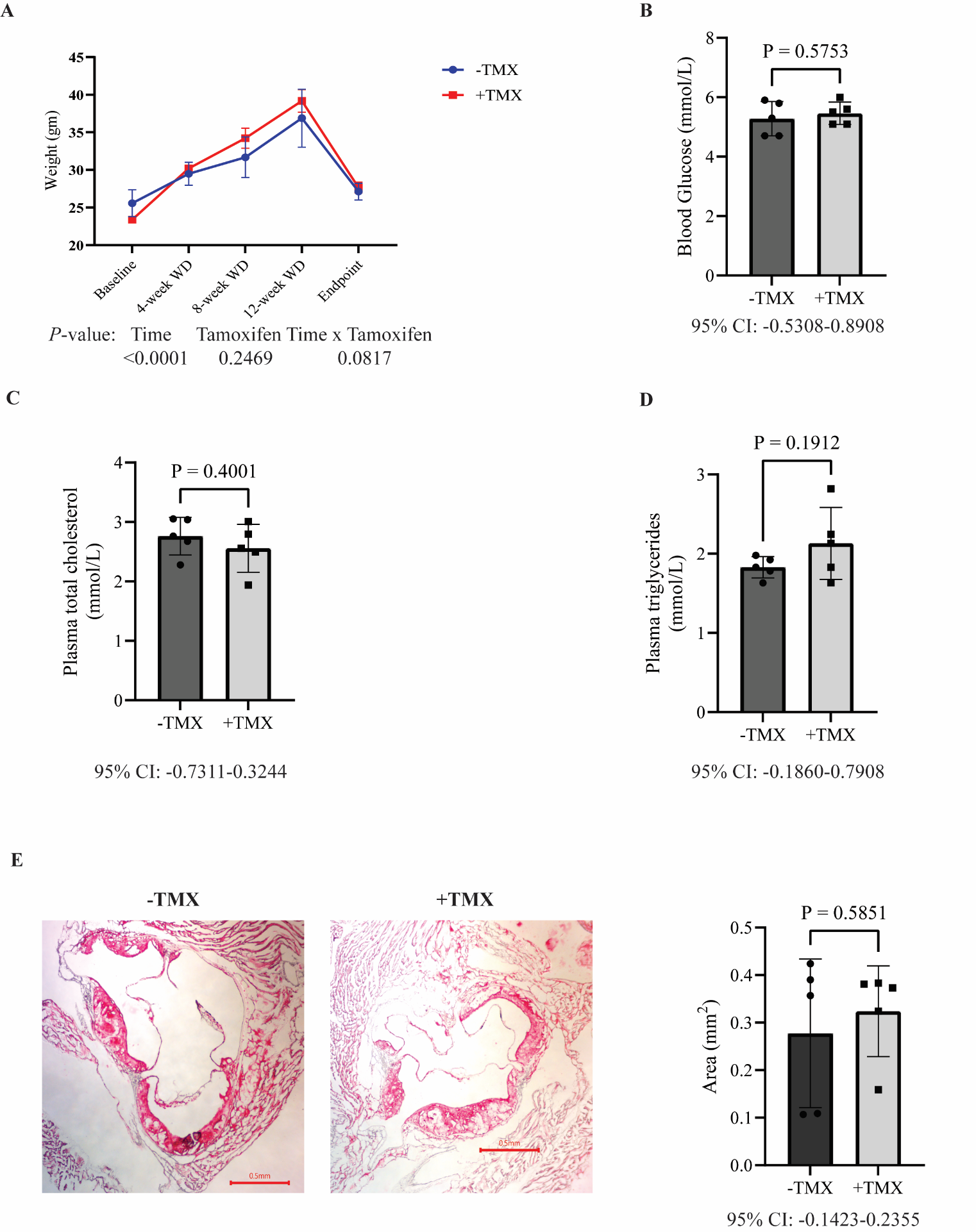
**Figure S16: Effect of tamoxifen on atherosclerosis regression. A and B,** Body weight (A) and blood glucose (B) of -TMX and +TMX mice (n=5 mice per group). **C and D,** Plasma total cholesterol (TC, C) and plasma triglycerides (TG, D) of -TMX and +TMX mice (n=5 mice per group). **E,** Representative pictures and quantification of oil red O-stained aortic sinus of -TMX and +TMX mice at endpoint (n=10 mice per group, scale bar= 0.5mm). *P*-value was calculated by linear mixed effects model with mouse ID included as a random effect (REML) in panel A, by unpaired two-tailed Student's t-test in panels B-E.

**
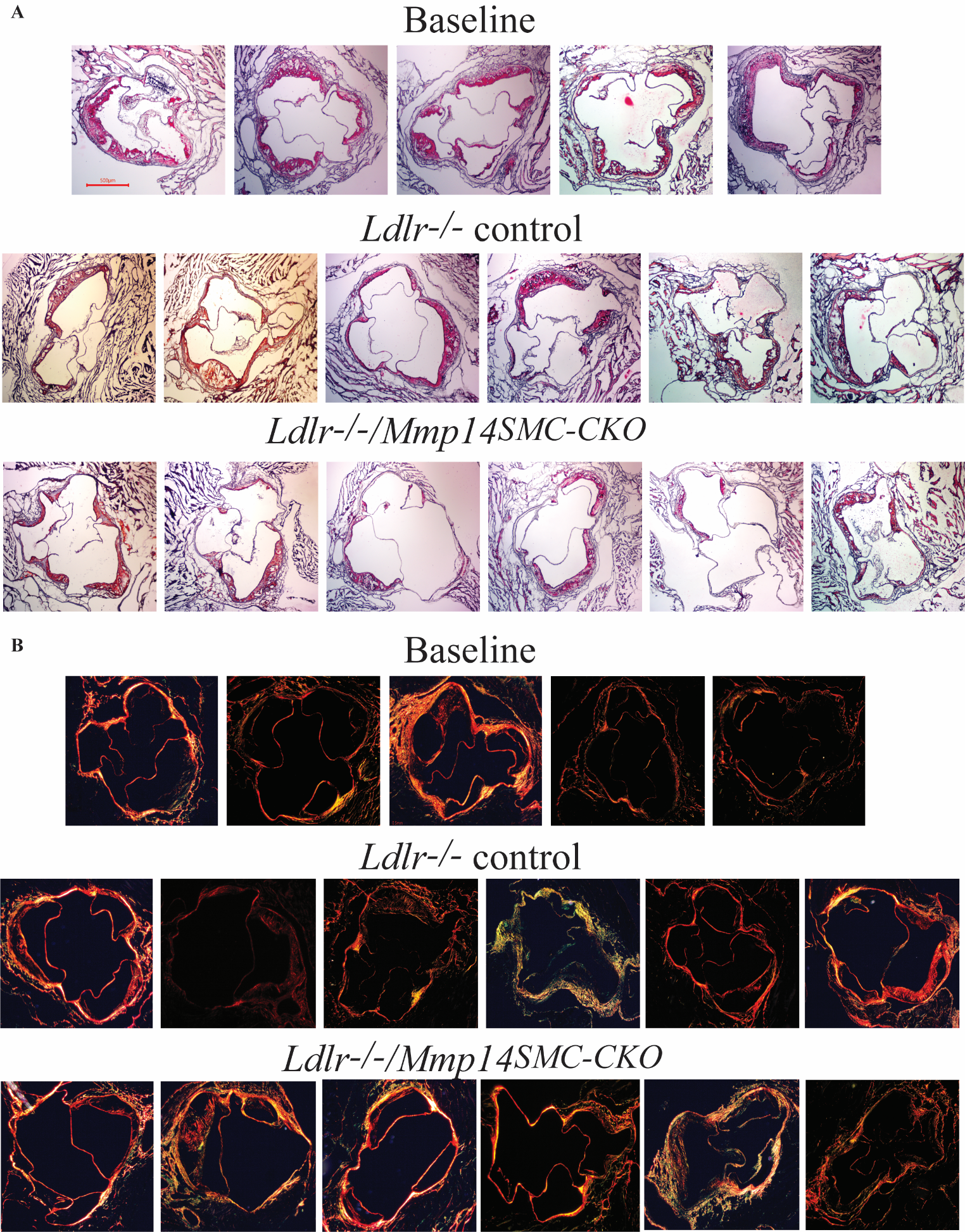
**

**Figure S17: Atherosclerosis regression in baseline, *Ldlr*^-/-^ control and *Ldlr*^-/-^/*Mmp14*^SMC-CKO^ mice**. Pictures of oil-red-O stained aortic sinus (A) and picrosirius red-stained aortic sinus (B) of baseline, *Ldlr*^-/-^ control and *Ldlr*^-/-^/*Mmp14*^SMC-CKO^ mice in the regression study (n=5-6/group).
